# The E3 Ligase APIP10 Connects the Effector AvrPiz-t to the NLR Receptor Piz-t in Rice

**DOI:** 10.1371/journal.ppat.1005529

**Published:** 2016-03-31

**Authors:** Chan Ho Park, Gautam Shirsekar, Maria Bellizzi, Songbiao Chen, Pattavipha Songkumarn, Xin Xie, Xuetao Shi, Yuese Ning, Bo Zhou, Pavinee Suttiviriya, Mo Wang, Kenji Umemura, Guo-Liang Wang

**Affiliations:** 1 Department of Plant Pathology, Ohio State University, Columbus, Ohio, United States of America; 2 State Laboratory for Biology of Plant Diseases and Insect Pests, Institute of Plant Protection, Chinese Academy of Agricultural Sciences, Beijing, China; 3 Biotechnology Research Institute, Fujian Academy of Agricultural Sciences, Fuzhou, Fujian, China; 4 Meiji Seika Kaisha Ltd, Health & Bioscience Laboratories, Tokyo, Japan; University of California, Davis Genome Center, UNITED STATES

## Abstract

Although nucleotide-binding domain, leucine-rich repeat (NLR) proteins are the major immune receptors in plants, the mechanism that controls their activation and immune signaling remains elusive. Here, we report that the avirulence effector AvrPiz-t from *Magnaporthe oryzae* targets the rice E3 ligase APIP10 for degradation, but that APIP10, in return, ubiquitinates AvrPiz-t and thereby causes its degradation. Silencing of *APIP10* in the non-*Piz-t* background compromises the basal defense against *M*. *oryzae*. Conversely, silencing of *APIP10* in the *Piz-t* background causes cell death, significant accumulation of Piz-t, and enhanced resistance to *M*. *oryzae*, suggesting that APIP10 is a negative regulator of Piz-t. We show that APIP10 promotes degradation of Piz-t via the 26S proteasome system. Furthermore, we demonstrate that AvrPiz-t stabilizes Piz-t during *M*. *oryzae* infection. Together, our results show that APIP10 is a novel E3 ligase that functionally connects the fungal effector AvrPiz-t to its NLR receptor Piz-t in rice.

## Author Summary

Rice is the staple food for half of the world’s population. Rice diseases are, however, the major threat for stable rice production worldwide. Elucidating the molecular basis is pivotal for the development of durable resistance to control rice diseases. We previously found that the RING finger E3 ligase APIP6 interacts with AvrPiz-t and plays a role in rice PAMP-triggered immunity (PTI). In this study, we characterized another RING finger E3 ligase in rice, named APIP10. Like APIP6, APIP10 and AvrPiz-t degrade each other, and APIP10 is a positive regulator of PTI. Interestingly, reduction of *APIP10* expression level in the *Piz-t* resistant plants causes severe cell death and accumulation of the NLR receptor Piz-t, indicating APIP10 is a negative regulator of Piz-t. We also show that APIP10 can promote Piz-t degradation while AvrPiz-t can stabilize Piz-t. Our results demonstrate that APIP10 is a target of a fungal effector and a negative regulator of an NLR receptor in plants.

## Introduction

Unlike animal responses to pathogen infection, plant responses to pathogen infection do not include a circulatory system or specialized cells [1]. Instead, individual plant cells launch defense responses against invading pathogens. Extensive molecular studies over the last two decades have revealed two layers of host immunity in plants. Plant immunity can be activated when highly conserved pathogen-associated molecular patterns (PAMPs) are recognized by plasma membrane-bound pattern recognition receptors (PRRs) in a process called PAMP-triggered immunity (PTI). PTI is considered the first layer of plant immunity [2,3]. For the second layer, immunity can be activated when pathogen-delivered avirulence (Avr) effectors are recognized by the product of plant resistance (R) genes in a process called effector-triggered immunity (ETI). ETI can be achieved by the direct or indirect interaction between the Avr effectors and R proteins in the plant cell [1,4]. Upon recognition, both immunity layers are capable of initiating a signaling cascade that can result in multiple defense responses.

The nucleotide-binding domain, leucine-rich repeat (NLR) proteins play a major role as intracellular immune receptor R proteins in plant immunity[5]. Most R genes cloned to date encode NLR proteins that mediate recognition of diverse effectors from all classes of plant pathogens. Both direct and indirect interactions between NLRs and effectors occur in different pathosystems [6]. In the indirect interactions, additional plant proteins are the targets of effectors and may be either genuine virulence targets of the effectors [7] or decoy proteins that plants have evolved to mimic *bona fide* effector targets [8]. A hybrid model of the direct and indirect interactions was proposed in which the target protein serves as ‘bait’ that the effector associates with before direct interaction with the NLR receptor and before immune signaling is activated [9,10].

Because NLR activation and signaling usually results in strong defense responses and a hypersensitive reaction (HR), such activation and signaling must be tightly regulated to avoid adverse effects on plant growth and development when plants are not under pathogen attack [11–13]. Some factors controlling NLR activation and signaling have been identified [10]. For example, analysis of crystal structure and *in vitro* interactions revealed that the two CHORD domains of a single RAR1 molecule bridge the N-termini of the HSP90 monomers, thus regulating the ‘open’ and ‘closed’ state of the HSP90 dimer that coordinates NLR stabilization [14]. Two independent studies showed that the tetratricopeptide repeat-containing protein SRFR1 is a negative regulator of the accumulation and activation of the NLR receptor SNC1 [15,16].

Ubiquitin-mediated degradation of proteins via the 26S proteasome is important for the regulation of protein levels in living cells [17]. The E3 ligases in the ubiquitination process interact and bring substrates to be ubiquitinated in proximity to the conjugating enzyme E2. Involvement of ubiquitination in NLR-mediated immunity has been recently reported in plants (see review by [18]). For example, the SCF E3 ubiquitin ligase complex involving the F-box protein CPR30/CPR1 (SCF^CPR1^) targets the NLR protein SNC1 and RPS2 for degradation [11,12]. Furthermore, when plants overexpressing CPR1 were treated with the 26S proteasomal inhibitor MG132, levels of SNC1 increased. This observation suggested that SNC1 levels are modulated by SCF^CPR1^ via the 26S proteasome-mediated degradation pathway. However, it is unknown whether these E3 ligase complexes are the host targets of pathogen effectors.

*Magnaporthe oryzae* is the causal agent of rice blast, a severe disease that limits rice production worldwide. We cloned the R gene *Piz-t* from rice and its cognate avirulence gene *AvrPiz-t* from *M*. *oryzae* [4,19]. *Piz-t* encodes an NLR protein, but *AvrPiz-t* does not show homology to any genes in the databases. Our previous study showed that AvrPiz-t is secreted into the rice cell and interacts with the rice E3 ligase APIP6 [20]. Analysis of *APIP6* RNAi transgenic plants showed that APIP6 is a positive regulator of PTI. AvrPiz-t and APIP6 can degrade each other *in planta*. APIP10 is another E3 ligase that was identified in the yeast-two hybrid screen when AvrPiz-t was used as the bait in our previous study [20]. To determine the role of APIP10 in rice immunity in the current study, we analyzed the relationships among APIP10, AvrPiz-t, and Piz-t. We report that APIP10 contributes to rice immunity by functioning in both PTI and ETI. While APIP10 is the target of AvrPiz-t’s virulence activity for suppression of PTI, APIP10 is also a negative regulator of the accumulation and activation of the NLR receptor Piz-t. Degradation of APIP10 by AvrPiz-t results in the release of APIP10 suppression of Piz-t accumulation and the initiation of a strong defense response. Our study identifies a novel E3 ligase, APIP10, that functionally connects a fungal effector with its NLR receptor in plants.

## Results

### AvrPiz-t interacts with the putative E3 ligase APIP10 *in vitro* and *in vivo*

We previously reported that AvrPiz-t interacts with three putative C3HC4-type E3 ligases in yeast-two hybrid (Y2H) screens [20]. Among the AvrPiz-t interacting proteins (APIPs), APIP10 showed a strong interaction and was selected for further studies. We confirmed the interaction between AvrPiz-t and APIP10 in yeast using four selection markers (S1A Fig). To further validate the interaction in yeast, we performed *in vivo* Co-IP (co-immunoprecipitation) experiments by co-expressing *GFP*:*AvrPiz-t*:*HA* and *Myc*:*APIP10* in *Nicotiana benthamiana* using the agroinfiltration method. Because the AvrPiz-t signal peptide is cleaved in the mature protein, we used the *AvrPiz-t* gene without its N-terminal signal peptide sequence to make constructs in the following experiments unless indicated otherwise. When the GFP:AvrPiz-t:HA fusion protein was immunoprecipitated (IP) from the plant extract using anti-HA IgG beads, the Myc:APIP10 proteins were detected in the immunocomplex of GFP:AvrPiz-t:HA with the anti-Myc antibody (S1B Fig, middle lane of the upper-right panel). As a control, no visible background signal was detected in the samples expressing only GFP:AvrPiz-t:HA (S1B Fig, the third lane of the upper-right panel) or Myc:APIP10 (S1B Fig, the first lane of the upper-right panel). These results indicate that AvrPiz-t interacts with APIP10 *in vivo*.

Since AvrPiz-t interacts with three putative C3HC4-type E3 ligases in the Y2H screens as previously reported [20], we decided to confirm whether that the interaction between AvrPiz-t and APIP10 is specific by including an unrelated E3 ligase, SPL11 [21], as an additional negative control in the Co-IP experiment. To facilitate the detection of SPL11 *in planta* as reported previously [22], mSPL11, which has three mutations in the U-box domain, was used in the experiment. Similar with the assay in S1B Fig, *GFP*:*AvrPiz-t*:*HA*, *Myc*:*APIP10* and *Myc*:*mSPL11* were co-expressed in *N*. *benthamiana*. When the GFP:AvrPiz-t:HA fusion protein was immunoprecipitated from the plant extract using the anti-HA IgG beads, the Myc:APIP10 proteins were also detected in the immunocomplex of GFP:AvrPiz-t:HA with the anti-Myc antibody (S1C Fig, first lane of the top-right panel). However, no visible signal was detected in the sample expressing only *GFP*:*AvrPiz-t*:*HA* (S1C Fig, third lane of the top-right panel) or co-expressing *GFP*:*AvrPiz-t*:*HA* and *Myc*:*mSPL11* (S1C Fig, second lane of the top-right panel). These results indicate that the interaction *in planta* between AvrPiz-t and APIP10 is specific.

### APIP10 is a functional RING finger E3 ligase

*APIP10* identified from the Y2H library consists of a 1,416-bp open reading frame and encodes a protein with 472 amino acids. BLAST searches showed that *APIP10* is a single-copy gene in the rice genome and that the predicted protein belongs to a large family of conserved and ubiquitous RING finger proteins in eukaryotes. APIP10 has multiple, predicted conserved domains such as a BRAP2 (BRCA1-Associated Protein 2), a C3HC4-type RING finger, a ZnF UBP (Zinc-Finger Ubiquitin Binding Protein), and a coiled-coil domain (S2A Fig).

To determine whether APIP10 has E3 ligase activity like other BRAP2-RING Finger-ZnF UBP proteins, we performed an *in vitro* ubiquitination assay with a series of negative controls. In the E3 ligase activity assay, the MBP:APIP10 fusion protein purified from *E*. *coli* was incubated with wheat (*Triticum aestivum*) E1, Arabidopsis E2 (AtUBC10), 5X Myc:ubiquitin (Myc:Ub), and ATP. To verify whether the E3 ligase activity of APIP10 depends on its RING finger domain, we included the mutant dRING (APIP10 dRING) in which the RING finger at the amino acid position from 158 to 204 was completely deleted. In the preliminary experiment, no polyubiquitin product was visible when Ub was used. Therefore, Ub fused with 5X Myc was used in the reaction, and the immunoblot with anti-Myc antibody was performed to enhance the detection of the polyubiquitin. A band of polyubiquitin product with high molecular weight was detected in the reaction with the complete APIP10 (S2B Fig, lane 6, arrow indicated) but not in the reaction with APIP10 dRING (S2B Fig, lane 5 in the top panel), suggesting that the RING finger domain is required for the E3 ligase activity of APIP10. To further confirm this result, we performed an E3 ligase assay with time and E2 enzyme quantity as variables and with APIP10 dRING as negative a control. The immunoblot analysis with anti-Myc antibody showed that a signal of the high molecular weight protein band (about 170 kD) became stronger as the time was increased (the protein band is indicated by an arrow in S3A Fig in the top panel). Similarly, the signal of the high molecular weight protein band was stronger when more E2 enzyme was added to the reaction (as indicated by an arrow in S3B Fig in the top panel).

### AvrPiz-t interferes with the E3 ligase activity of APIP10 and is ubiquitinated by APIP10 *in vitro*

**Fig 1:**
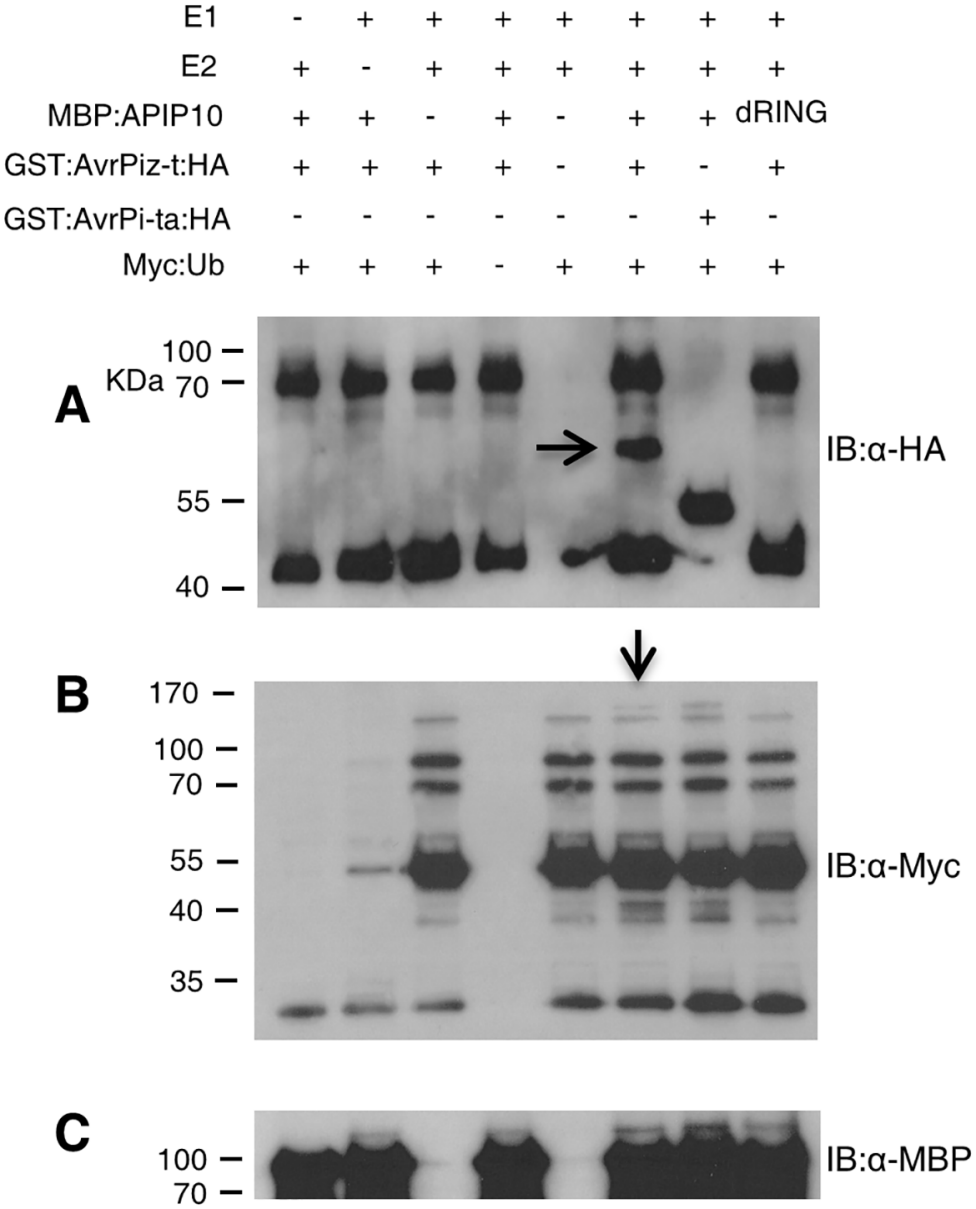
Ubiquitination of AvrPiz-t by APIP10 and suppression of APIP10 E3 ubiquitin ligase activity by AvrPiz-t *in vitro*. (**A**) *In vitro* ubiquitination assay of GST:AvrPiz-t:HA by MBP:APIP10 fusion protein. Ubiquitination of AvrPiz-t by APIP10 was detected by immunoblot with the anti-HA antibody. GST:AvrPi-ta:HA was used as a negative control to determine the specificity of AvrPiz-t ubiquitination by APIP10. The experiments were performed at least three times with similar results. (**B**) Suppression of the APIP10 E3 ubiquitin ligase activity by AvrPiz-t. E3 activity of APIP10 in the presence of AvrPiz-t or AvrPi-ta was determined by immunoblot with the anti-Myc antibody. (**C**) Immunoblot with anti-MBP antibody to determine the amount of MBP-APIP10 or MBP protein loaded in each lane.

To assess whether AvrPiz-t has any biochemical functions in the APIP10-mediated ubiquitination, we included the purified GST:AvrPiz-t:HA protein in the *in vitro* E3 ligase assay of APIP10. Surprisingly, a band above the GST:AvrPiz-t:HA protein was detected by the immunoblot analysis with the anti-HA antibody in the presence of the GST:AvrPiz-t:HA recombinant protein (Fig 1A, lane 6, arrow). In contrast, no such signal was detected in the presence of the GST:AvrPi-ta:HA protein, an unrelated effector protein from *M*. *oryzae* [23] (Fig 1A, lane 7). The difference in size between the two bands is about the size of a 5X Myc:ubiquitin (about 16 KDa), indicating that the GST:AvrPiz-t:HA is ubiquitinated by APIP10 *in vitro*.

To confirm the ubiquitination result, we repeated the E3 ligase assay for the reactions in lane 6–8 in Fig 1A. Then we conducted a GST pulldown analysis by washing the GST:AvrPiz-t:HA and GST:AvrPi-ta:HA beads with the PBST solution (Phosphate Buffered Saline with 0.5% Triton X-100) after the E3 ligase reaction. Immunoblot analysis with the anti-HA antibody showed the ubiquitinated bands above the GST:AvrPiz-t:HA fusion protein (S4 Fig, first lane in the upper panel). Immunoblot analysis with the anti-ubiquitin antibody showed a smear of high molecular bands above GST:AvrPiz-t:HA only in the reaction with both the wild type MBP:APIP10 and GST:AvrPiz-t:HA (S4 Fig, first lane in lower panel), but not with both MBP:APIP10 and GST:AvrPi-ta:HA (S4 Fig, second lane in the lower panel) or with both MBP:APIP10 dRING and GST:AvrPiz-t:HA (S4 Fig, third lane in the lower panel). These results demonstrated that AvrPiz-t is specifically ubiquitinated by APIP10 *in vitro*.

Furthermore, the immunoblot analysis that used the anti-Myc antibody to detect polyubiquitin showed that the E3 ligase activity of APIP10 was reduced when the GST:AvrPiz-t:HA fusion protein was included in the reaction (Fig 1B, lane 6, arrow), suggesting that AvrPiz-t interferes with APIP10 E3 ligase activity. The GST:AvrPi-ta:HA protein, which did not affect the E3 ligase activity of APIP6 [20], was used as a negative control (Fig 1B, lane 7). These results show that AvrPiz-t interferes with APIP10 E3 ligase activity and that APIP10 ubiquitinates AvrPiz-t *in vitro*.

### APIP10 and AvrPiz-t promote degradation of each other through the 26S proteasome system in *N*. *benthamiana*

**Fig 2:**
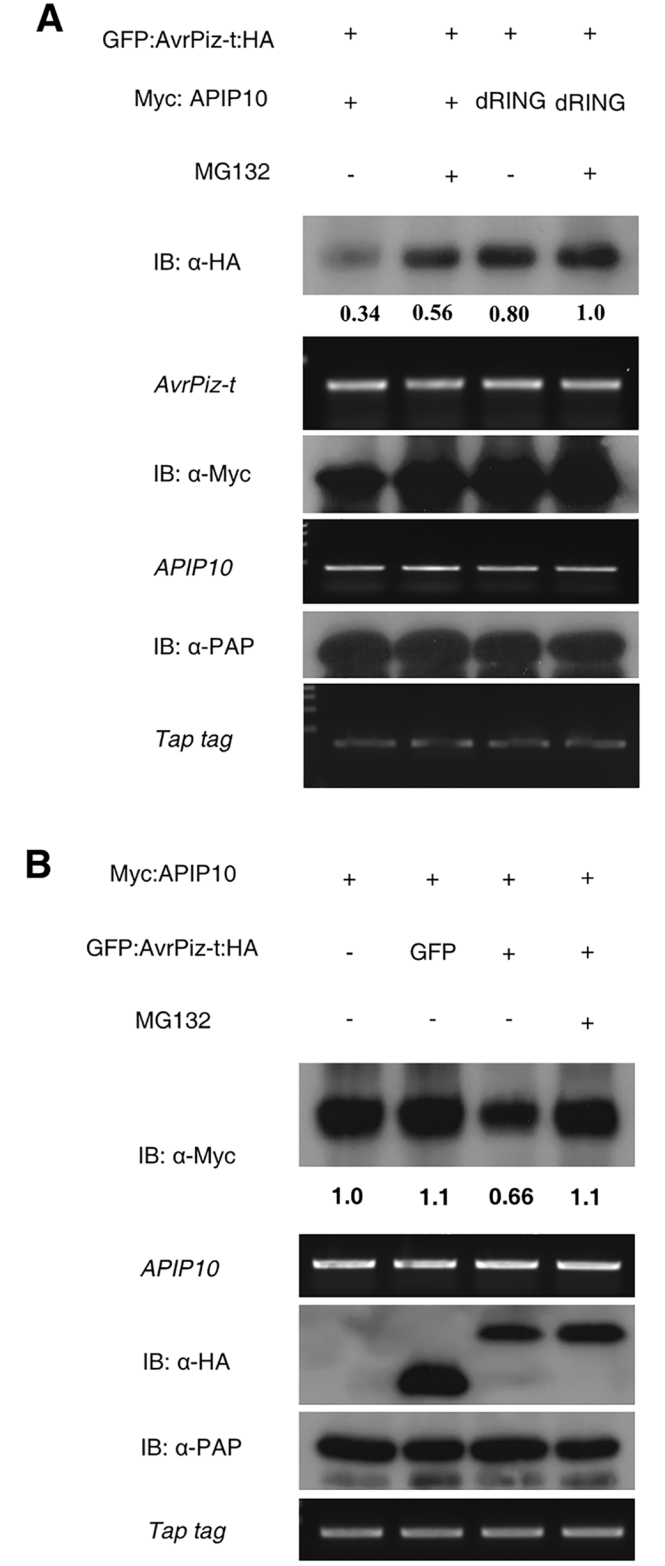
AvrPiz-t and APIP10 degrade each other in *N*. *benthamiana*. (**A**) Degradation of GFP:AvrPiz-t:HA with co-expression of Myc:APIP10 in *N*. *benthamiana*. Myc:APIP10 or Myc:APIP10 dRING was co-expressed with GFP:AvrPiz-t:HA by agro-infiltration. Tissues were harvested 2 days after infiltration. MG132 (50 μM) was infiltrated with DMSO as a control at 18 h before sampling. Tap tag protein was expressed as an internal control and was detected by immunoblot with the peroxidase anti-peroxidase (PAP). The transcriptional level of each gene was determined by semi-quantitative (sq)-PCR. dRING denotes APIP10 dRING. The experiments were performed at least three times with similar results. (**B**) Degradation of APIP10 when it is co-expressed with the AvrPiz-t protein in *N*. *benthamiana*. Tissues were harvested 3 days after infiltration. MG132 (50 μM) was infiltrated with DMSO as a control at 18 h before sampling. The experiments were performed at least three times with similar results.

Because AvrPiz-t is ubiquitinated by APIP10 *in vitro* (Fig 1A), we hypothesized that AvrPiz-t may be a substrate of APIP10 *in planta*. To test this, we co-expressed *GFP*:*AvrPiz-t*:*HA* with *Myc*:*APIP10* in *N*. *benthamiana* leaves. The immunoblot analysis revealed that the GFP:AvrPiz-t:HA protein level was significantly lower in the tissue when *Myc*:*APIP10* was co-expressed than when *Myc*:*APIP10 dRING* was co-expressed (Fig 2A, compare lane 1 and 3 in the top panel). This result suggests that APIP10 promotes the degradation of AvrPiz-t in plant cells. Pre-treatment of the leaves with MG132 inhibited the degradation of GFP:AvrPiz-t:HA (Fig 2A, lane 2 in the first panel), suggesting that APIP10 degrades AvrPiz-t via the 26S proteasome system. To test whether the mutated APIP10 dRING affects the stability of its associated protein AvrPiz-t, we used Myc:GFP as a negative control and repeated the co-infiltration assay. The western blot analysis revealed that GFP:AvrPiz-t:HA was significantly lower in the tissue where APIP10 was co-expressed (S5 Fig, first lane in the top panel) compared to the control where Myc:GFP was co-expressed (S5 Fig, third lane in the top panel). This result confirmed that that APIP10 promotes the degradation of AvrPiz-t in plant cells.

Because AvrPiz-t promotes the degradation of APIP6 when they are co-expressed in *N*. *benthamiana* [20], we reasoned that AvrPiz-t might also affect the accumulation of APIP10 in plant cells. The immunoblot analysis showed that APIP10 was reduced by 30–40% (relative to the controls) when GFP:AvrPiz-t:HA and Myc:APIP10 were co-expressed (Fig 2B, lane 3 in the upper panel). Furthermore, the degradation was inhibited by MG132 (Fig 2B, lane 4 in the upper panel), suggesting that AvrPiz-t promotes the degradation of APIP10 *in planta*, likely through the 26S proteasome system. To obtain more evidence that the degradation of APIP10 is dependent of AvrPiz-t, AvrPii, an unrelated effector protein [24], was used as a negative control in the co-infiltration. The immunoblot analysis showed that the accumulation of Myc:APIP10 was significantly decreased by the co-expression with GFP:AvrPiz-t:HA (. S6 Fig, first lane in the top panel) compared to the negative control, co-expression of Myc:APIP10 with GFP:AvrPii:HA (S6 Fig, third lane in the top panel). This provides additional evidence that the degradation of APIP10 *in planta* is dependent on AvrPiz-t.

### Knockdown of *APIP10* in the NPB background (*Piz-t* ^*-*^) compromises PTI

**Fig 3:**
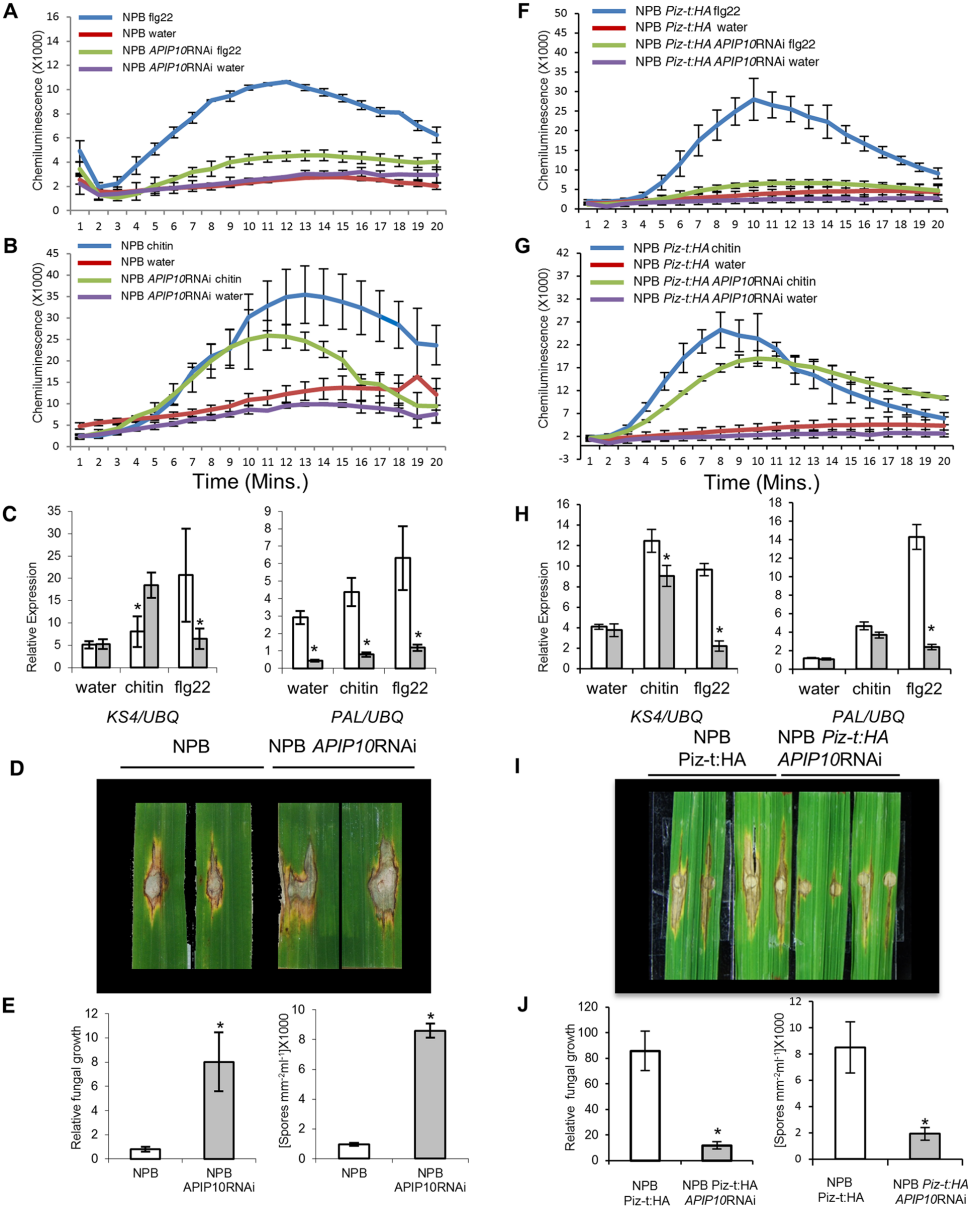
Knockdown of *APIP10* expression compromises basal defense in rice. (**A**), (**B**) PTI-induced reactive oxygen species (ROS) burst in three independently transformed NPB *APIP10* RNAi and NPB lines. The mean of the three lines was used for data analysis. (**F**), (**G**) PTI-induced ROS burst in NPB *Piz-t*:*HA* and NPB *Piz-t*:*HA APIP10* RNAi lines. Rice leaf disks were treated with 100 nM flg22, 8 nM chitin (*hexa-N*-acetyl-chitohexaose), or water. ROS were detected using a luminol-chemiluminescence assay. Error bars, s.e.m. (*n* = 3). (**C**), (**H**) Induction of defense-related genes *OsKS4* and *OsPAL* at 3 h post incubation (hpi) in either water, chitin, or flg22, respectively; white bars indicate NPB lines in C and NPB *Piz-t*:*HA* in H, and grey bars indicate *APIP10* RNAi plants in respective backgrounds. qPCR was performed using gene-specific primers. Values are means and error bars, s.e.m. (*n* = 3). (**D**), (**I**) Infection assay: rice leaves of 6-week-old plants were inoculated with the virulent isolate RB22; the leaves were photographed 9 d post inoculation (dpi). (**E**), (**J**). Relative fungal growth (left) and sporulation (right) were measured 9 dpi. Values are means, error bars are s.e.m. (*n =* 8, *P<0.05)

As described above, AvrPiz-t interferes with the E3 ligase activity of APIP10 *in vitro* (Fig 1B) and promotes the degradation of APIP10 *in vivo* (Fig 2B). Based on these results and our previous report on APIP6 [20], we speculated that APIP10 might be another target of AvrPiz-t-mediated suppression of host defense. To determine the role of APIP10 in PTI against *M*. *oryzae*, we designed an RNAi construct targeting 215 bp of *APIP10* 3’UTR using the pCXUN vector [25] and generated over 20 stable transgenic lines in NPB (without *Piz-t*). Three homozygous lines with a single T-DNA insertion were selected from the T_3_ generation for the subsequent analysis and transcription levels of each line were determined by qRT-PCR (S7 Fig, top panel). To determine the function of *APIP10* in rice PTI, we used a luminol-based chemi-luminescence assay to monitor ROS generation induced by flg22 and chitin treatments in 4-week-old homozygous *APIP10* RNAi plants [26]. The analysis showed that *APIP10* knockdown significantly suppressed flg22-induced ROS accumulation (Fig 3A) but only partially suppressed chitin-induced ROS accumulation in the rice tissue (Fig 3B). At 3 h after treatment with flg22 and chitin, qRT-PCR also revealed that the transcriptional profiles of PTI-related defense genes such as *KS4* and *PAL* differed between *APIP10* RNAi plants and non-silenced plants (Fig 3C). *KS4* encodes one of the two diterpene cyclase enzymes involved in momilactone biosynthesis [27,28], and the *APIP10* RNAi plants showed significant suppression of *KS4* transcripts 3 h after treatment with flg22 (Fig 3C, left). Phenylalanine ammonia lyase (PAL) catalyzes the deamination of L-phenylalanine to *trans-*cinnamic acid and is involved in the biosynthesis of certain classes of low molecular weight antimicrobial compounds called phytoalexins [29,30]. In rice, the transcription of *PAL* is induced by flagellin from bacteria as well as by chitin [31–33]. In *APIP10* RNAi lines, significant suppression of *PAL* transcripts was observed at 3 h after treatment with either flg22 or chitin (Fig 3C, right). Suppression of *PAL* transcripts without PAMP treatment was also observed (Fig 3C, water), indicating that *APIP10* might be involved in PAMP-independent regulation of *PAL*. Taken together, these results suggest that host immunity triggered by PAMPs is compromised in *APIP10* RNAi plants.

Next, we used the punch inoculation method to measure the resistance of *APIP10* RNAi plants to the virulent *M*. *oryzae* isolate RB22. Lesions were larger on the *APIP10* RNAi plants than on the control plants (Fig 3D). The relative fungal mass of *M*. *oryzae*, measured by the DNA-based qPCR assay, was also greater in the *APIP10* RNAi plants than in the control plants (Fig 3E, left panel). In addition, more spores were produced on the *APIP10* RNAi plants than on the control plants (Fig 3E, right panel). These results suggest that silencing of the *APIP10* gene in rice compromises the basal defense against *M*. *oryzae*. Because the phenotypes of *APIP10* RNAi lines are similar to those of APIP6 RNAi lines, we conducted qRT-PCR of *APIP6* in the *APIP10* RNAi lines (.S8A Fig, top panel) and *APIP10* in the *APIP6* lines (S8B Fig, lower panel). The analysis showed that there is no correlation between the expression levels of the two genes when one of them is silenced.

### Knockdown of *APIP10* in the *Piz-t* background causes severe spontaneous cell death phenotypes

**Fig 4:**
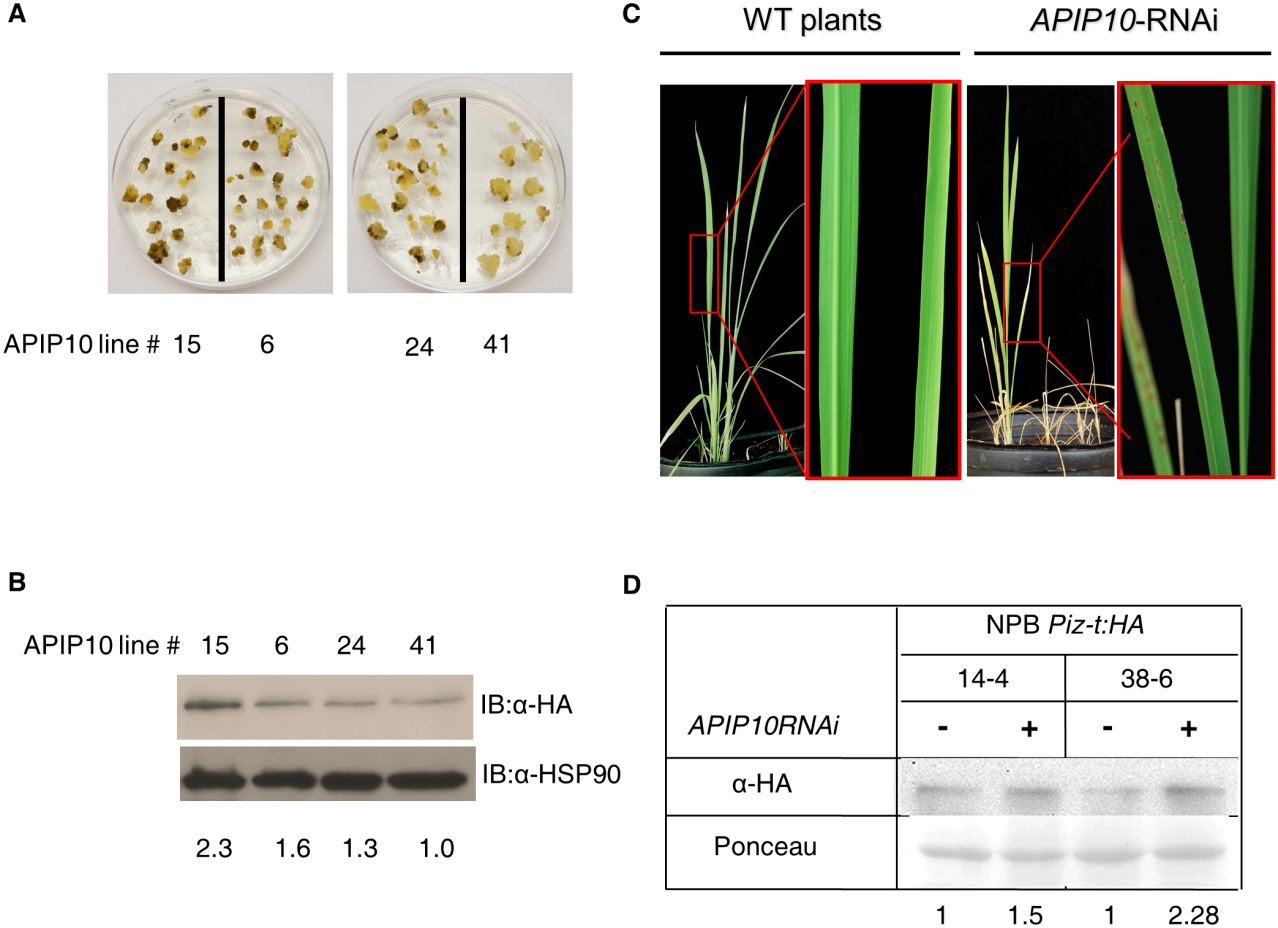
APIP10 negatively regulates Piz-t:HA accumulation. **(A**) Cell death phenotype of *APIP10* RNAi T0 callus lines in NPB-*Piz-t*:*HA* background. (**B**) Western blot with the *APIP10* RNAi Callus lines in NPB Piz-t:HA background. Western blot was conducted with the anti-HA body to detect the level of the Piz-t proteins. The HSP90 protein was used to check the loading level. Numbers below the panel are the level of Piz-t measured with ImageJ software and normalized by the amount of HSP90. (**C**) Phenotype of the *APIP10* RNAi lines with strong cell death. (**D**) Immunoblots with the anti-HA antibody indicating levels of Piz-t:HA in segregated WT NPB *Piz-t*:*HA* and *APIP10*-silenced NPB *Piz-t*:*HA* plants. Ponceau staining of the blot indicates total protein loading. Numbers below the panel are Piz-t:HA band intensities normalized by Ponceau staining of Rubisco.

*Piz-t* is the cognate R gene of *AvrPiz-t* in rice [4]. To understand the relationship between APIP10 and Piz-t, we first transformed the *Piz-t*:*HA* construct with hygromycin as a selection marker in the NPB background in order to generate NPB *Piz-t*:*HA* rice. Then we screened T1 lines for hygromycin resistance and chose the lines showing a 3:1 segregation ratio. After the T_2_ plants were inoculated with the avirulent isolate RO1-1, the presence of the Piz-t:HA fusion protein was confirmed by immunoblot analysis with the anti-HA antibody. With this approach, we obtained five homozygous lines with a single T-DNA insertion of *Piz-t*:*HA*. Next, we developed an *APIP10* RNAi construct (same as described above) with the G418 Sulfate antibiotic selection and transformed the construct into the calli of selected NPB *Piz-t*:*HA* line. The *APIP10* transcription levels in selected lines were determined by qRT-PCR (S7 Fig, lower panel). Surprisingly, NPB *Piz-t*:*HA* calli transformed with the *APIP10* RNAi construct showed severe cell death but not with an empty vector (Fig 4A and S9 Fig).

To determine whether the severe cell death triggered in the calli is related to the Piz-t:HA protein, we performed immunoblot analysis for Piz-t:HA with the anti-HA antibody and qRT-PCR of the *APIP10* transcripts; we did this with four independently transformed callus lines that showed different levels of cell death. qRT-PCR indicated that the transcript level of *APIP10* was lower in lines with severe cell death than in the line without cell death (S10 Fig, the upper panel), indicating that the cell death observed in the transgenic plants was caused by the silencing of *APIP10*. Strikingly, the lines with lower *APIP10* transcripts in the NPB *Piz-t*:*HA* background showed a higher expression level of the Piz-t-HA protein (Fig 4B, the upper panel), suggesting that the *APIP10* transcript level is negatively correlated with Piz-t accumulation and cell death. To determine whether the accumulation of the Piz-t protein is due to its increased transcription, we used qRT-PCR to quantify *Piz-t* transcripts. The analysis revealed that the number of *Piz-t* transcripts in the *APIP10* RNAi callus lines was inversely related to Piz-t protein accumulation (.S10 Fig, the lower panel), indicating that the accumulation of the Piz-t protein is not the result of its increased transcription.

After two rounds of rice transformation, we were able to obtain only six T1 lines that had reduced cell death because most *APIP10* RNAi lines died within 2–3 weeks after transfer to soil (Fig 4C, right panel). To confirm the results obtained from callus lines, we compared the T_2_ plants of the *APIP10* RNAi lines in the NPB *Piz-t*:*HA* background with the wild-type NPB *Piz-t*:*HA* plants. All of the lines in the NPB *Piz-t*:*HA* background showed cell death and dwarf phenotypes (S11A Fig). Immunoblot analysis of total protein obtained from the leaf tissue of two lines (#14–4 and 38–6) revealed that they contained 1.5- to 2.2-times more Piz-t:HA than the controls (Fig 4D). qRT-PCR analysis using the same leaf tissue showed that *Piz-t* transcripts were less abundant in the *APIP10* RNAi lines 14–4 and 38–6 than in the control plants (S11B Fig), indicating that the Piz-t accumulation is not due to an increase in its transcription level.

### Knockdown of *APIP10* in the *Piz-t* background compromises PTI but still enhances resistance to a virulent isolate

To determine whether PTI responses of *APIP10* RNAi lines in NPB *Piz-t*:*HA* were compromised as we had observed in the *APIP10* RNAi lines in NPB, we monitored the ROS generation triggered by chitin and flg22 in the leaf disks of 4-week-old plants. The analysis showed that silencing of *APIP10* in the presence of *Piz-t* almost completely suppressed ROS induced by flg22 in the leaf tissue (Fig 3F) but did not suppress ROS induced by chitin significantly (Fig 3G). This observation was similar to that observed in the *APIP10* silencing lines in NPB (Fig 3B). qRT-PCR also revealed that the RNAi lines showed significant but distinct transcriptional induction or suppression profiles of the PTI-related defense genes 3 h after treatment with chitin or flg22 (Fig 3H). For example, the *APIP10* RNAi lines showed suppression of *KS4* transcripts at 3 h after treatment with flg22 or chitin (Fig 3H, left) but showed significant suppression of *PAL* transcripts at 3 h after treatment with flg22 but not after treatment with chitin (Fig 3H, right).

We next used the punch inoculation method to measure the resistance of the *APIP10* RNAi lines in the NPB *Piz-t*:*HA* background to the virulent *M*. *oryzae* isolate RB22. Surprisingly, lesions were smaller on the *APIP10* RNAi plants in the *Piz-t* background than on the wild-type NPB-*Piz-t*:*HA* plants (Fig 3I); this was opposite to the disease phenotype observed in the *APIP10* RNAi plants in the NPB background (Fig 3D). Consistent with lesion size, the relative fungal biomass measured by the DNA-based qPCR was significantly lower in the *APIP10* RNAi-NPB-*Piz-t*:*HA* plants than in the *Piz-t*:*HA* plants (Fig 3J, left panel). Furthermore, the sporulation in the infected area was significantly lower on the *APIP10* RNAi-NPB-*Piz-t*:*HA* plants than on the *Piz-t*:*HA* plants (Fig 3J, right panel). Taken together, these results suggest that silencing of *APIP10* in the *Piz-t* plants enhances resistance to the virulent isolate RB22 possibly through the accumulation of Piz-t, even though the PTI response was compromised because of the silencing of *APIP10*.

### APIP10 promotes degradation of the Piz-t protein through the 26S proteasome system

Because we found that silencing of *APIP10* in transgenic rice leads to accumulation of the Piz-t protein (Fig 4B and 4D), we speculated that *APIP10* may promote the degradation of Piz-t in rice. To test this hypothesis, we co-expressed either *Myc*:*APIP10* or *Myc*:*APIP10 dRING* (as a negative control) with *Piz-t*:*HA* using agroinfiltration in *N*. *benthamiana* leaves. Less Piz-t:HA protein accumulated when it was co-expressed with APIP10 than when it was co-expressed with APIP10 dRING, and the accumulation was recovered almost to the control level by treatment with MG132 (S12 Fig, lane 1 vs. 2 and lane 3 vs. 4 in the first panel).

**Fig 5:**
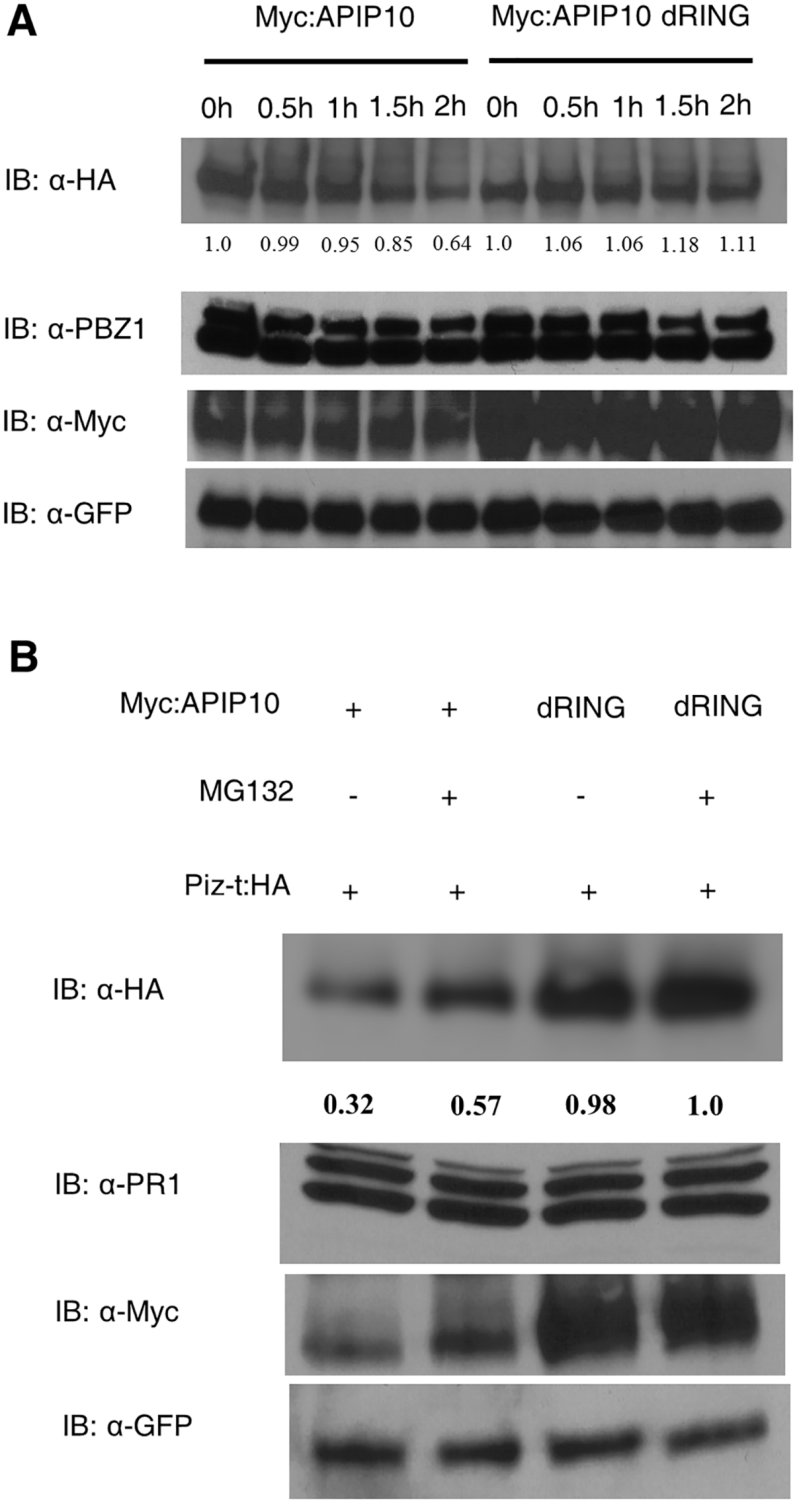
Semi-*in vivo* degradation of Piz-t:HA by APIP10 through the 26S proteasome system. (**A**) Degradation of Piz-t by APIP10 depends on APIP10 E3 ligase activity. Total protein from the inoculated rice plants was mixed with total protein from *N*. *benthamiana* in which either *Myc*:*APIP10* or *Myc*:*APIP10 dRING* (dRING) was co-expressed with *GFP*. PBZ1 or GFP was used as a loading control for Piz-t and Myc:APIP10 or Myc:dRING, respectively. (**B**) Suppression of Piz-t degradation by MG132 treatment. As in Fig 2A, MG132 was added to inhibit the degradation of proteins through the 26S proteasome system; the reaction was stopped at 4 h after incubation. PR1 or GFP was used as a loading control for Piz-t:HA and Myc:APIP10 or Myc:APIP10 dRING (dRING), respectively.

To confirm these results, a semi-*in vivo* degradation assay was performed as described previously [34,35]. For this assay, total protein extracted from the *M*. *oryzae*-inoculated *Piz-t*:HA rice plants was mixed with total protein extracted from *N*. *benthamiana* leaves co-expressed with either *APIP10* or *APIP10* dRING with GFP as an internal control. The samples were collected at different times before 1X SDS loading buffer was added to stop the reaction. The level of Piz-t was decreased by APIP10 over time, and after 2 h, less Piz-t protein was detected in the presence of APIP10 (Fig 5A, lane 5) than in the presence of APIP10 dRING (Fig 5A, lane 10). The decrease in the level of the Piz-t protein at 4 h was partially inhibited by treatment with MG132 (Fig 5B, lane 2 in the first panel). To rule out the possibility that the deletion of the RING finger domain in APIP10 stabilizes both APIP10 dRING and Piz-t, myc:GFP was used as a negative control instead of APIP10 dRING in the semi *in vivo* degradation assay. The decrease in the level of the Piz-t protein was observed in the presence of Myc:APIP10 (S13 Fig, lane 1 vs. lane 3 in the top panel) and the degradation of Piz-t:HA was partially inhibited by the MG132 treatment (S13 Fig, lane 1 vs. lane 2). However, no obvious degradation of Piz-t in the presence of Myc:GFP was observed (S13 Fig, lane 3 vs. lane 4 in the top panel). Together, these data suggest that APIP10 promotes Piz-t degradation through the 26S proteasome system.

### AvrPiz-t promotes the accumulation of the Piz-t protein *in planta*

**Fig 6:**
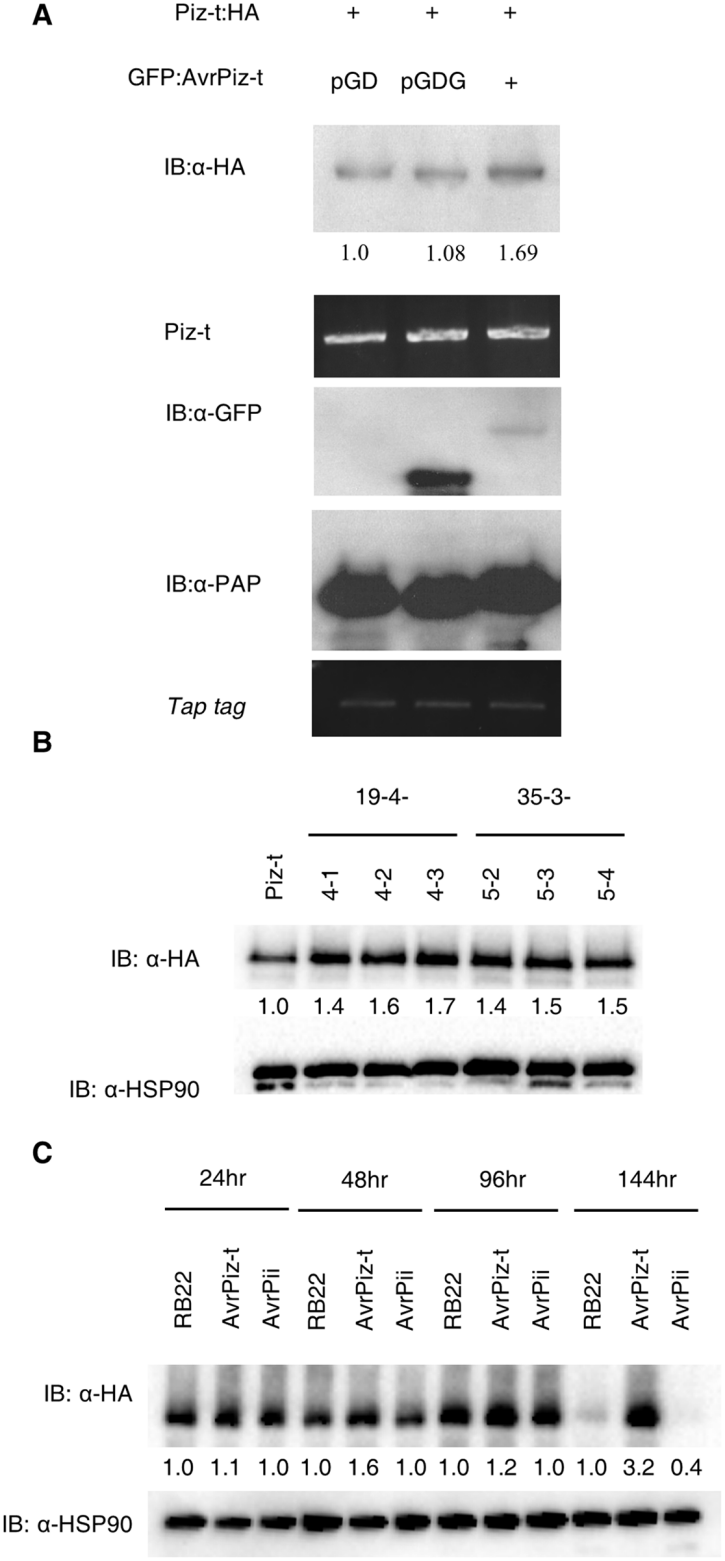
AvrPiz-t promotes the accumulation of Piz-t protein *in planta*. (**A**) Co-expression of *Piz-t*:*HA* with *GFP*:*AvrPiz-t*:*HA* in *N*. *benthamiana*. *Piz-t*:*HA* was co-expressed either with pGD, pGDG (GFP), or *GFP*:*AvrPiz-t*. The native promoter in the *Piz-t* genomic construct was replaced by the 35S promoter to express the *Piz-t* gene in *N*. *benthamiana*. Tissues were harvested at 3 days after agroinfiltration. As negative controls, the vectors pGD and pGDG were expressed. TAP tag was expressed as an internal control to check the efficiency of agroinfiltration. (**B**) Immunoblot with anti-HA antibody was used to indicate levels of Piz-t after estradiol-mediated induction of *AvrPiz-t* in NPB Piz-t:HA plants. The leaf samples were collected 24 h after the estradiol treatment. The numbers below the immunoblot indicate relative band intensity. HSP90 was used as a loading control. (**C**) Immunoblot with anti-HA antibody was used to indicate levels of Piz-t at different time points after inoculation in the NPB Piz-t:HA plants infected with *M*. *oryzae* isolate RB22, RB22:*AvrPiz-t* and RB22:*AvrPii*. The number below each sample is the relative intensity compared to that of RB22-inoculated sample at each time point. HSP90 was used as a loading control.

Because we found that AvrPiz-t promotes degradation of APIP10 and that silencing of APIP10 leads to accumulation of Piz-t, we reasoned that expression of AvrPiz-t *in planta* may lead to the accumulation of Piz-t. To determine the relationship between AvrPiz-t and Piz-t, we co-expressed either empty vector, *GFP*, or *GFP*:*AvrPiz-t* with *Piz-t*:*HA* in *N*. *benthamiana* and observed the accumulation of both proteins by immunoblot analysis. Intriguingly, the Piz-t protein level was ~1.6-fold greater when *Piz-t*:*HA* was co-expressed with GFP:AvrPiz-t:HA (Fig 6A, top panel, lane 3) than with the empty vector or with GFP (Fig 6A, top panel, lane 1 and 2), suggesting that AvrPiz-t stabilizes the Piz-t protein. To determine whether the increased accumulation of Piz-t in the presence of AvrPiz-t is specific to AvrPiz-t, we included an unrelated effector protein from *M*. *oryzae*, AvrPii, as a negative control. As described above, we co-expressed the empty pGD vector, *GFP*:*AvrPiz-t or GFP*:*AvrPii* with *Piz-t*:*HA* in *N*. *benthamiana* and observed the accumulation of proteins by immunoblot analysis. The assay showed that the Piz-t protein level was ~2.7-fold greater when *Piz-t*:*HA* was co-expressed with GFP:AvrPiz-t:HA (S14 Fig, the middle lane in the top panel) than that with the empty vector or with GFP:AvrPii (S14 Fig, the first and the third lanes in the top panel, respectively), suggesting that the stabilization of Piz-t by AvrPiz-t is specific.

To confirm this result in rice plants, we transformed the construct expressing the *AvrPiz-t* gene under the control of the inducible XVE system into the rice calli generated from the *Piz-t*:*HA* rice seeds in order to generate *iAvrPiz-t* transgenic lines. Over 20 independently transformed transgenic lines were obtained. After confirming the genotype by PCR, we selected six T_4_ lines with the *Piz-t* plants as a negative control for the following analyses. Young leaves of each line were cut and transferred to N6 medium containing the β-estradiol to induce the expression of *AvrPiz-t*. Piz-t accumulation increased upon the induction of *AvrPiz-t* (Fig 6B). The induction of *AvrPiz-t* as well as the level of *Piz-t* transcript was monitored by qRT-PCR after the treatment as shown in S15 Fig

To confirm the above results with rice plants under physiologically relevant conditions, i.e., when rice plants were inoculated with *M*. *oryzae*, we inoculated the *Piz-t* plants by the spray method with RB22 isogenic blast transformants: RB22, RB22:*AvrPiz-t*, and RB22:*AvrPii*. Consistent with the results from *N*. *benthamiana*, Piz-t:HA accumulation was greater in the RB22:*AvrPiz-t*-inoculated plants than in the RB22 or RB22:*AvrPii*-inoculated plants at all the time points we monitored (Fig 6C). The Piz-t level was still high in the RB22:*AvrPiz-t*-inocualted plants at 144 h after inoculation, confirming that AvrPiz-t promotes the accumulation of the Piz-t protein *in planta*.

## Discussion

In host–microbe interactions, ubiquitination plays an important role in both host defense and pathogen infection [18,36]. Researchers have identified both pathogen effectors that target the ubiquitination machinery for defense suppression and plant ubiquitination-related proteins that target pathogen effectors for degradation and thus for defense enhancement. Most of these findings, however, have been derived from several model plant diseases caused by bacterial pathogens. Although recent research has revealed the importance of ubiquitination in plant diseases caused by fungi or oomycetes [20,37], the role of ubiquitination in these diseases is poorly understood. In particular, how microbial perturbation of host ubiquitination activates the defense response mediated by NLR receptor proteins is largely unknown. In this study, we describe the relationship among the fungal effector AvrPiz-t, the rice RING finger E3 ligase APIP10, and the NLR receptor Piz-t. We found that AvrPiz-t can interact with and promote the degradation of APIP10 when it is secreted into rice cells by *M*. *oryzae*. Silencing of *APIP10* in the *Piz-t* background causes severe cell death and accumulation of Piz-t. Co-infiltration assays showed that APIP10 expression leads to Piz-t degradation and that such degradation depends on the RING finger domain of APIP10. In contrast, AvrPiz-t can stabilize Piz-t during blast infection. These results demonstrate an elegant defense mechanism in which rice cells use the E3 ligase APIP10 to regulate Piz-t for immune responses when attacked by the blast fungus carrying the *AvrPiz-t* gene. Degradation of the APIP10 by AvrPiz-t and the resulting elimination of the negative regulation of Piz-t protein by APIP10 leads to a rapid increase in Piz-t and a strong defense response including programmed cell death in the infected cells. Although APIP10 seems similar to RIN4 in *Arabidopsis* in that they both negatively regulate an NLR [7], the ability of APIP10 to degrade a pathogen effector (AvrPiz-t) and to regulate a host NLR (Piz-t) is unique. Therefore, identification of the novel E3 ligase APIP10 that functionally connects a fungal effector to its cognate NLR receptor in rice provides new insights into plant innate immunity.

We found that AvrPiz-t interacts with APIP10 and that this interaction reduces APIP10 E3 ligase activity. APIP10 specifically ubiquitinates AvrPiz-t but not the unrelated effector AvrPi-ta. The ubiquitination of AvrPiz-t depends on the RING domain of APIP10, and the degradation of AvrPiz-t depends on the 26S proteasome system. These results suggest that AvrPiz-t could be the pseudo-substrate of APIP10. By acting as the pseudo-substrate, AvrPiz-t might prevent APIP10 from ubiquitinating either the host proteins or even other cytoplasmic effector proteins secreted from the fungus. Interaction with AvrPiz-t reduces APIP10’s E3 ligase activity and the stability of APIP10. Turnover of E3 ligase enzymes that contain RING domains is regulated in many ways in cells. For example, CBL RING E3 ligases have substrate-dependent regulation in animal systems because substrate specificity plays a role in CBL turnover [38–40]. Turnover of the rice E3 ligase APIP10 by a fungal effector is an interesting phenomenon. Further investigation of the mechanism underlying APIP10 degradation by AvrPiz-t in rice may provide new insights into fungal–plant interactions.

Ubiquitination is important in the regulation of defense-related NLR proteins in plants. Recent research shows that SCF^CPR1^ regulates the levels of SNC1 and RPS2 [11,41]. Also, COP1-dependent regulation of an NLR HRT was observed in response to *turnip crinkle virus* [13]. In this study, we found that APIP10 is a negative regulator of the NLR receptor Piz-t. Specifically, Piz-t levels are higher in the *APIP10* RNAi plants than in control plants, which is consistent with the accumulation of Piz-t after infection with isolates carrying AvrPiz-t. The accumulation of the Piz-t protein after the recognition of AvrPiz-t is unique because the R proteins RPM1 and RPG1 disappear after the recognition of cognate effectors [42,43]. RPM1 is rapidly degraded with effector-mediated activation, perhaps due to a negative feedback that limits HR and attenuates the disease resistance response [42]. The same laboratory reported that the disappearance of activated RPM1 is not dependent on the proteasome system because the two common proteasome inhibitors, MG132 and clasto-lactacystin β-lactone, were not able to stabilize steady-state levels of RPM1 [44]. Our results show that the Piz-t protein level is regulated by the proteasome system. However, we don’t know whether the accumulation of the Piz-t protein due to reduced transcription levels of *APIP10* leads to the activation of Piz-t and whether the activated form is translocated into the nucleus. In addition, the accumulation of Piz-t in *N*. *benthimiana* when AvrPiz-t is expressed is intriguing because it implies that *N*. *benthamiana* may have functional otholog(s) of APIP10. Indeed, two APIP10 homologs (Niben101Scf01072g01009.1, 47% identity, and Niben101Scf05720g06002.1, 43% identity) were found in the Sol Genomics Network Database (https://solgenomics.net/).

In addition, the APIP10 RING domain is vital for Piz-t degradation, suggesting the importance of E3 ligase activity in regulating NLRs. Even though the negative regulation of Piz-t by APIP10 during *M*. *oryzae* infection is clear, how APIP10 regulates the Piz-t protein level before and after *M*. *oryzae* infection remains unknown. Because no direct interaction between APIP10 and Piz-t was found in yeast, we speculate that a partner or chaperone protein links APIP10 and Piz-t in rice cells. Identifying this unknown protein is one of our objectives in dissecting the Piz-t-mediated signaling pathway.

**Fig 7:**
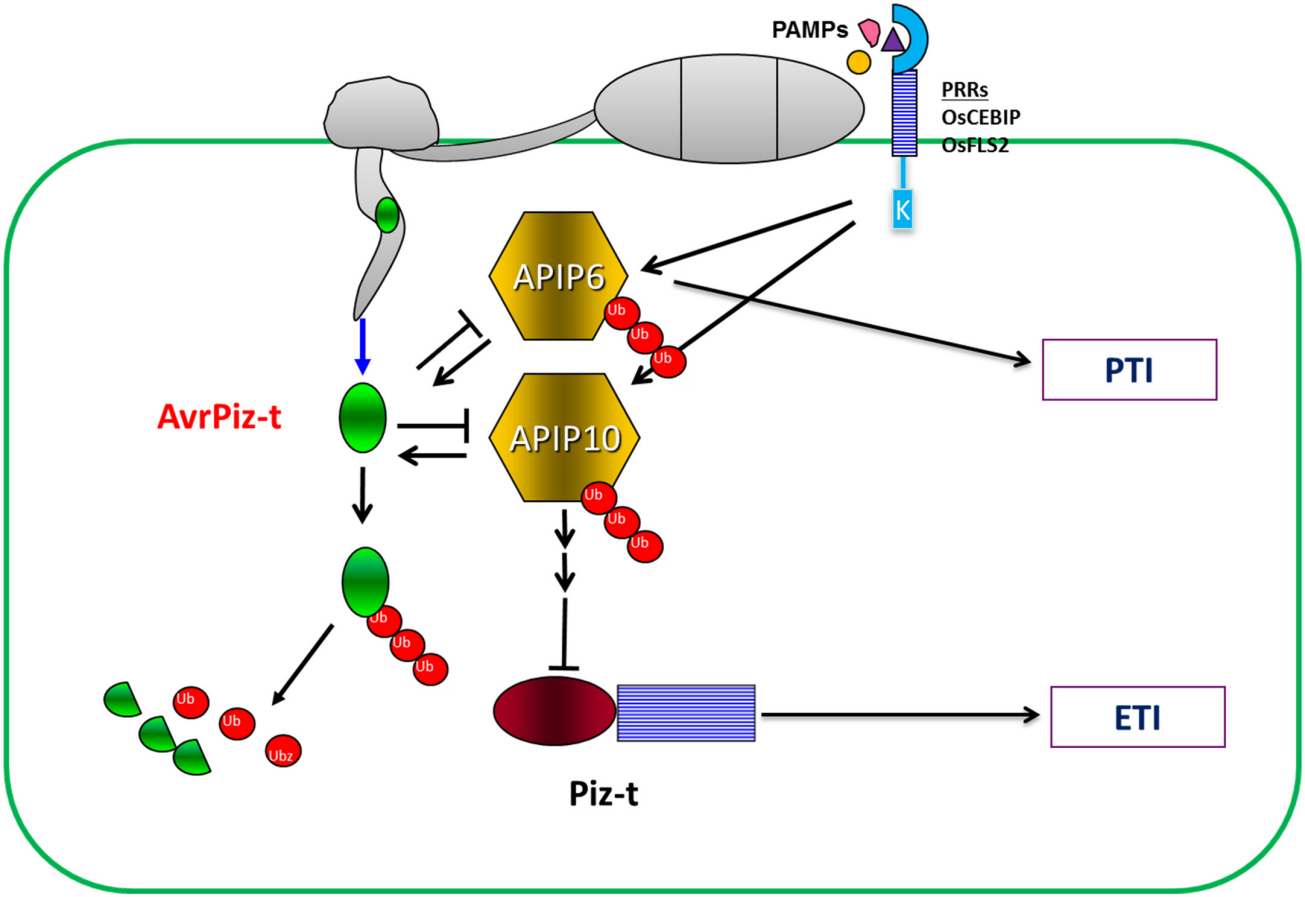
Working model of the relationships among AvrPiz-t, APIP6, APIP10, and Piz-t. When *M*. *oryzae* spores land on a rice leaf and germinate, cell-surface localized PRRs can recognize PAMPs, which leads to PTI as a defense mechanism against the fungal infection. To counteract PTI, *M*. *oryzae* secretes effector proteins including AvrPiz-t. Once AvrPiz-t enters rice cells, it is translocated into rice cells and promotes the degradation of APIP6/10, which are positive regulators of PTI, to suppress defense responses such as accumulation of ROS and induction of defense-related genes. However, APIP10 is also a negative regulator of Piz-t in rice cells, and the degradation or modification of APIP10 by AvrPiz-t removes the negative regulation on Piz-t and thus leads to the accumulation of Piz-t, which triggers ETI-mediated responses. To counter its degradation by AvrPiz-t, APIP10 ubiquitinates AvrPiz-t, which leads to the degradation of the effector in the rice cell.

Many Avr/R protein-protein interactions fit into the guard model, but the molecular mechanism that regulates the recognition and signaling between the pathogen and host proteins is incompletely understood, especially for fungal and oomycete pathogens of plants. In addition and as noted earlier, the regulation of NLR receptors before and after pathogen infection is still unclear. In our previous study [20], we found that AvrPiz-t targets the E3 ligase APIP6 for degradation and that APIP6 in turn degrades AvrPiz-t and positively regulates PTI to *M*. *oryzae* in rice. In this study, we found that APIP10 not only has a similar relationship as APIP6 with AvrPiz-t but also negatively regulates the NLR protein Piz-t via ubiquitination. Based on our previous results and those from this study, we provide the following model to explain the relationships among AvrPiz-t, APIP6, APIP10, and Piz-t (Fig 7). After being secreted and translocated into rice cells, AvrPiz-t interacts with both APIP6/10 and interferes with their E3 ligase activity. In response, APIP6/10 ubiquitinate AvrPiz-t, which causes the degradation of AvrPiz-t in rice cells. When AvrPiz-t is delivered into rice cells without Piz-t, it promotes the degradation of APIP6/10 to suppress PTI. When the *Piz-t* plants are not attacked by *M*. *oryzae*, APIP10 directly or indirectly maintains the Piz-t protein at a low level through ubiquitination. However, when AvrPiz-t is delivered into rice cells expressing Piz-t, AvrPiz-t interacts with and degrades APIP10, which removes the negative regulation on Piz-t and causes the rapid accumulation of Piz-t protein. The rapid accumulation of Piz-t triggers a strong HR and the activation of defense responses.

## Materials and Methods

### Agroinfiltration assay in *N*. *benthamiana*

*Agrobacterium tumefaciens* strain GV3101 carrying different constructs was grown with shaking at 28°C. After 18 h, bacterial cells were spun down for 20 min at 3,200 *g* and then resuspended in MES buffer (10 mM MgCl_2_ and 10 mM MES, pH 5.6) to a final OD_600_ of 1.5 for testing constructs, OD_600_ of 1.0 for *p19*, and OD_600_ of 0.25 for *TAP* tag. After acetosyringone was added to a final concentration of 150 μM, bacterial suspensions were kept at room temperature in the dark for 3 h and were then infiltrated into *N*. *benthamiana* plants as previously described [45].

### Rice plant induction treatment, inoculation, and gene expression analysis

The punch inoculation method [46] with a slight modification was used to evaluate infection of rice plants by *M*. *oryzae*. Isolate RB22 was cultured on oat meal agar medium for 2 weeks. A 10-μl volume of a spore suspension (5 × 10^5^ spores ml^-1^) was applied to slightly punctured sites of leaves on plants that were 4 to 6 weeks old. Lesion diameter was recorded 10 days after inoculation. To measure the sporulation rate, we removed a 3×1 cm^2^ leaf piece that included a lesion and immersed it in a microcentrifuge tube containing 100 μl of distilled water with 1% Tween 20. The samples were vigorously mixed in a vortex apparatus for 2 min to dislodge the spores, and the number of spores ml^-1^ was determined with a microscope and hemacytometer. The infection ratio was calculated as previously described [47]. Student’s *t*- test was used to test the significance of the differences between the segregating wild-type and lines expressing the transgenes.

qRT-PCR was used to determine gene expression in rice plants or detached leaves. After treatments had been applied to plants or detached leaves, total RNAs were extracted from leaf tissue using TRIzol reagent (Invitrogen) according to the manufacturer’s instructions. Total RNA was treated with DNase I (Invitrogen) to remove DNA contamination following the manufacturer’s protocol. About 1 μg of DNase I-treated RNA was subjected to first-strand cDNA synthesis using the Promega Reverse Transcription System (Promega). qRT-PCR was carried out using the iQ5 real-time PCR detection system (Bio-Rad). Primers used in this study are listed in S1 Table.

### Measurement of ROS level

The second leaf from the top of 4- to 6-week-old rice plants was used for the measurement of ROS. Small leaf discs (approximately 4 mm in diameter) were cut from the leaves with a cork borer and pre-incubated overnight in sterile-distilled water. After the leaf disks were treated with elicitors, ROS generation was monitored by the luminol chemi-luminescence assay [26]. Three pre-incubated leaf disks per sample were immersed in a microcentrifuge tube containing 100 μl of luminol (Bio-Rad), 1 μl of horseradish peroxidase (Jackson ImmunoResearch), and the elicitor (100 nM flg22, 8 nM hexa-N-acetylchitohexaose, or water control). Luminescence was measured at 10-s intervals for 21 min using a Glomax 20/20 luminometer (Promega). Each treatment was represented by three replicate microcentrifuge tubes.

### Semi-*in vivo* degradation of Piz-t:HA by APIP10

Semi-*in vivo* degradation of Piz-t:HA protein by APIP10 was measured as described before [34]. Briefly, total rice protein extracted from *M*. *oryzae*-inoculated *Piz-t*:*HA* plants was mixed with total protein from either *APIP10*- or *APIP10* dRING-agroinfiltrated *N*. *benthamiana* leaves. The reaction was started by adding 10 μM ATP to the mixture on ice. At the indicated time point, SDS loading buffer was added to the samples, which were boiled for 5 min to stop the reaction before immunoblot was performed.

More detailed experimental methods can be found in S1 Methods.

## Supporting Information

S1 Fig AvrPiz-t interacts with APIP10 *in vitro* and *in vivo*. (**A**) Y2H assays between BD-AvrPiz-t and AD-APIP10. Cells were plated on -Leu-Trp-His media containing 50mM 3-amino-1,2,4-triazole (3-AT), a competitive inhibitor of the His3p enzyme. (**B**) Co-immunoprecipitation (Co-IP) analysis of Myc:APIP10 and GFP:AvrPiz-t:HA *in planta*. GFP:AvrPiz-t:HA and Myc:APIP10 proteins were expressed in *N*. *benthamiana* using agro-infiltration. With preliminary experiments, the level of each protein was determined and normalized to the similar amount because we always observed less GFP:AvPiz-t and Myc:APIP10 when they are co-expressed compared to two controls. Co-IP experiment was performed with the anti-HA antibody and the protein was analyzed by western blot using the anti-Myc antibody and anti-HA antibody to detect APIP10 and AvrPiz-t, respectively. Asterisk indicates IgG band from immunoprecipitating the anti-HA antibody. (**C**) Co-IP analysis of Myc:APIP10 and GFP:AvrPiz-t:HA *in planta* using Myc:mSPL11 as a negative control. All conditions were the same as described in B.
(TIF)

S2 Fig Structure and E3 ubiquitin ligase activity assay of APIP10. (**A**) Protein structure of APIP10. BRCA1-Associated Protein 2), RING finger, a C3HC4 type zinc-finger, and ZnF UBP, Zinc-Finger Ubiquitin Binding Protein. (**B**) E3 ubiquitin ligase assay of APIP10. MBP:APIP10 fusion protein was assayed for E3 Ubiquitin ligase activity in the presence of Arabidopsis E1 (At5g06460), E2 (AtUBC10, At5g53300) and 5X Myc:ubiquitin. Either MBP:APIP10 dRING (lane 5) or MBP (lane 7) was used as a negative control. Immunoblot was performed with the anti-myc antibody to detect the polyubiquitin bands. Anti-His antibody used to detect E1, E2 and ubiquitin used in the reaction. A polyubiquitin band is indicated with an arrow.
(TIF)

S3 Fig E3 ubiquitin ligase activity assay of APIP10. **(A**) Time-course assay of E3 ubiquitin ligase activity of APIP10. Time course ubiquitin ligase assay was performed with APIP10 for 90 min. dRING was included as a negative control. The protein amount of either APIP10 or APIP10 dRING (dRING) loaded in each lane was determined by western blot with anti-MBP antibody. (**B**) E3 ubiquitin ligase activity assay of APIP10 with different amount of E2 enzyme. E3 ubiquitin ligase assay was performed with APIP10 for 90 min with different amount of AtUBC10 (At5g53300) enzyme as indicated. A polyubiquitin band is indicated with an arrow.
(TIF)

S4 Fig Confirmation of AvrPiz-t ubiquitination by APIP with GST pulldown. *In vitro* ubiquitination assay of GST:AvrPiz-t:HA by MBP:APIP10 was conducted with GST:AvrPiz-t:HA bound to glutathione agarose beads and washed five times with 1X PBST. Ubiquitinated GST:AvrPiz-t:HA was detected by western blot with the anti-HA antibody (upper panel) and the anti-Ub antibody after GST pulldown (lower panel).
(TIF)

S5 Fig Degradation of GFP:AvrPiz-t:HA with co-expression of Myc:APIP10 in *N*. *benthamiana*. Myc:GFP was used as a negative control instead of Myc:APIP10 dRING. G, Myc:GFP. Experimental conditions were the same as those described in Fig 2A
(TIF)

S6 Fig Degradation of GFP:AvrPiz-t:HA with co-expression of Myc:APIP10 in *N*. *benthamiana*. GFP:AvrPii:HA, an unrelated effector protein from *M*. *oryzae*, was used as a negative control. Tissues were harvested 3 days after infiltration. MG132 (50 μM) was infiltrated with DMSO as a control at 18 h before sampling. Experimental conditions were the same as those described in Fig 2B.
(TIF)

S7 Fig Transcript levels of *APIP10* in *APIP10RNAi* plants used for Fig 3. The transcript level of the ubiquitin (UBQ) was used for normalization. The analysis was repeated three times with similar results.
(TIF)

S8 Fig qRT-PCR of APIP10 in APIP6 RNAi lines and APIP6 in APIP10 RNAi lines. (**A**) Transcript levels of *APIP6* and *APIP10* in three different *APIP10* RNAi lines. (**B**) Transcript levels of *APIP6* and *APIP10* in three different *APIP6* RNAi lines
(TIF)

S9 Fig Cell death phenotype of APIP10 RNAi T0 Callus lines in NPB Piz-t:HA background. pCambia2300 empty vector was transformed into NPB Piz-t:HA background as a control of cell death phenotype observed in *APIP10* silencing callus lines.
(TIF)

S10 Fig Transcript levels of *APIP10* and *Piz-t* measured by qRT-PCR in *APIP10* RNAi NPB *Piz-t* callus lines. The expression level of *APIP10* and *Piz-t* was relative to that of the rice ubiquitin (UBQ) gene.
(TIF)

S11 Fig Stunted growth of *APIP10* RNAi NPB *Piz-t* lines and transcript levels of *APIP10* and *Piz-t* measured by qRT-PCR in 6-week old *APIP10* RNAi NPB *Piz-*t plants. (**A**) Stunted growth of the NPB *Piz-t*:*HA* plants when *APIP10* was silenced. (**B**) Transcript levels of *APIP10* and *Piz-t* measured by qRT-PCR in 6-week old NPB *Piz-t*:*HA APIP10RNAi* plants. *The* transcript level of the ubiquitin (UBQ) was used for normalization. Data represent means and error bars indicate s.e.m. (* p<0.05 and # p<0.05; *n* = 3).
(TIF)

S12 Fig Degradation of Piz-t:HA with co-expression of the Myc:APIP10 protein in *N*. *benthamiana*. *Piz-t*:*HA* was co-expressed either with *Myc*:*APIP10* or *Myc*:*APIP10 dRING* (dRING) in *N*. *benthamiana*. Agro-infiltrated tissues were harvested at 3 days after the infiltration and MG132 was treated 18 h before sampling. TAP tag was used as an internal control to normalize the agro-infiltration efficiency. Transcriptional level of each gene expression was determined by sqRT-PCR.
(TIFF)

S13 Fig Semi-*in vivo* degradation of Piz-t:HA by APIP10 through the 26S proteasome system. Degradation of Piz-t by APIP10 depends on APIP10 E3 ligase activity. Total protein from the inoculated rice plants was mixed with total protein from *N*. *benthamiana* in which either *Myc*:*APIP10* or *Myc*:*GFP* was co-expressed with *Taptag*. PBZ1 or Taptag was used as a loading control for Piz-t and Myc:APIP10 or Myc:GFP, respectively.
(TIF)

S14 Fig AvrPiz-t promotes the accumulation of Piz-t protein *in planta*. Co-expression of *Piz-t*:*HA* with *GFP*:*AvrPiz-t*:*HA* in *N*. *benthamiana*. The native promoter in the *Piz-t* genomic construct was replaced by the 35S promoter to express the *Piz-t* gene in *N*. *benthamiana*. *Piz-t*:*HA* was co-expressed either with pGD, *GFP*:*AvrPiz-t*, *or GFP*:*AvrPii*. Tissues were harvested at 4 days after agroinfiltration. TAP tag was expressed as an internal control to check the efficiency of agroinfiltration.
(TIF)

S15 Fig Transcript levels of *AvrPiz-t* (A) and *Piz-t*:*HA* (B) in the plants in which *AvrPiz-t* was induced by β-estradiol treatment. The samples were taken 24 h after the treatment. qRT-PCR with gene specific primer was performed, Ubiquitin (*UBQ)* transcript levels were used for normalization. Data are means of expression ratio and the error bars represent the s.e.m. (n = 2, P<0.05).
(TIF)

S1 Table Primers used in this study. (DOCX)

S1 Methods (DOCX)
