# Supplementary figures and images for: The E3 Ligase APIP10 Connects the Effector AvrPiz-t to the NLR Receptor Piz-t in Rice
Source: PLoS Pathog. 2016 Mar 31;12(3):e1005529. doi: 10.1371/journal.ppat.1005529 (PMC4816579; doi:10.1371/journal.ppat.1005529)

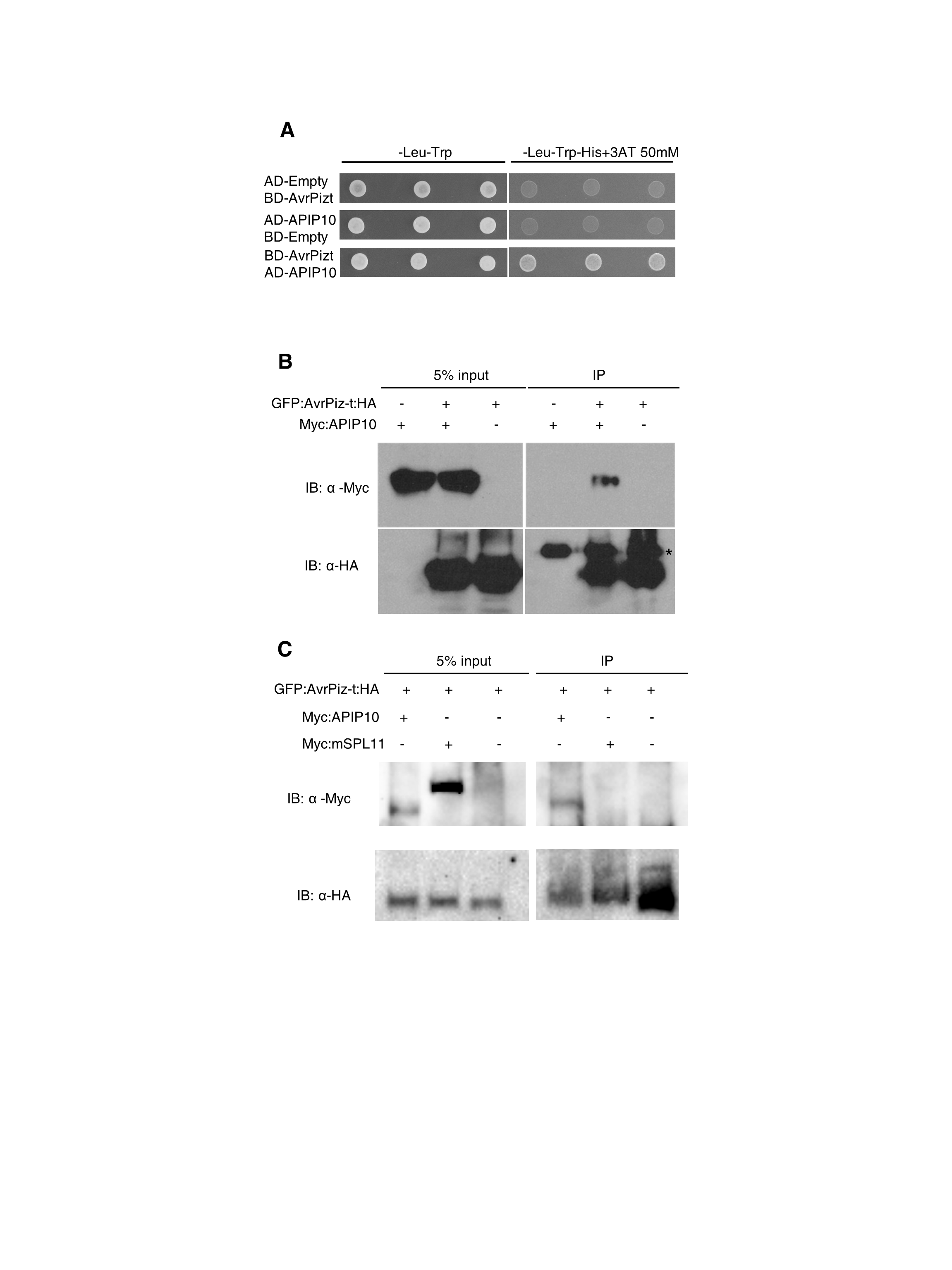

Supplement: S1 Fig — (A) Y2H assays between BD-AvrPiz-t and AD-APIP10. Cells were plated on -Leu-Trp-His media containing 50mM 3-amino-1,2,4-triazole (3-AT), a competitive inhibitor of the His3p enzyme. (B) Co-immunoprecipitation (Co-IP) analysis of Myc:APIP10 and GFP:AvrPiz-t:HA in planta. GFP:AvrPiz-t:HA and Myc:APIP10 proteins were expressed in N. benthamiana using agro-infiltration. With preliminary experiments, the level of each protein was determined and normalized to the similar amount because we always observed less GFP:AvPiz-t and Myc:APIP10 when they are co-expressed compared to two controls. Co-IP experiment was performed with the anti-HA antibody and the protein was analyzed by western blot using the anti-Myc antibody and anti-HA antibody to detect APIP10 and AvrPiz-t, respectively. Asterisk indicates IgG band from immunoprecipitating the anti-HA antibody. (C) Co-IP analysis of Myc:APIP10 and GFP:AvrPiz-t:HA in planta using Myc:mSPL11 as a negative control. All conditions were the same as described in B. (TIF) [file ppat.1005529.s001.TIF]

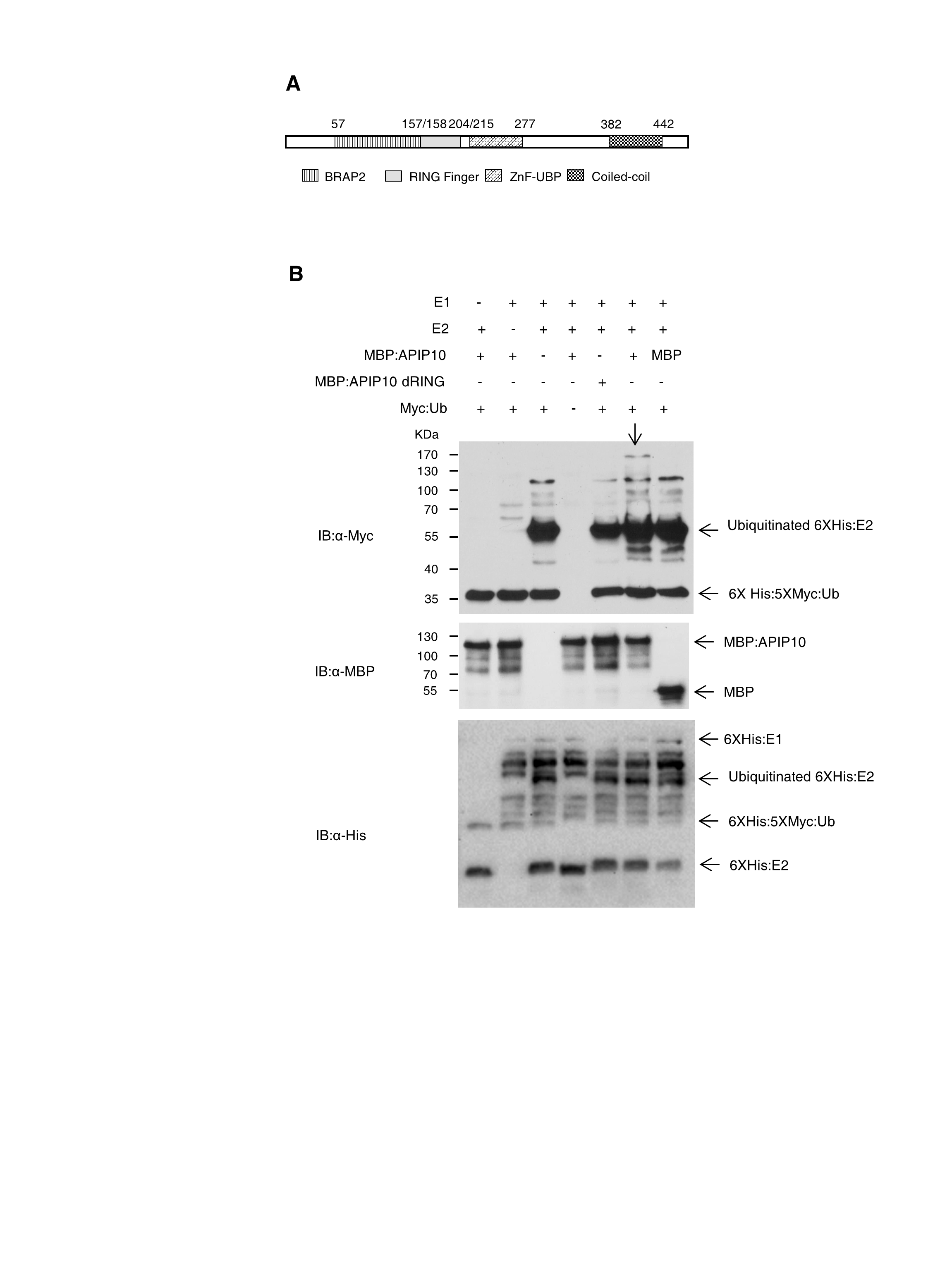

Supplement: S2 Fig — (A) Protein structure of APIP10. BRCA1-Associated Protein 2), RING finger, a C3HC4 type zinc-finger, and ZnF UBP, Zinc-Finger Ubiquitin Binding Protein. (B) E3 ubiquitin ligase assay of APIP10. MBP:APIP10 fusion protein was assayed for E3 Ubiquitin ligase activity in the presence of Arabidopsis E1 (At5g06460), E2 (AtUBC10, At5g53300) and 5X Myc:ubiquitin. Either MBP:APIP10 dRING (lane 5) or MBP (lane 7) was used as a negative control. Immunoblot was performed with the anti-myc antibody to detect the polyubiquitin bands. Anti-His antibody used to detect E1, E2 and ubiquitin used in the reaction. A polyubiquitin band is indicated with an arrow. (TIF) [file ppat.1005529.s002.TIF]

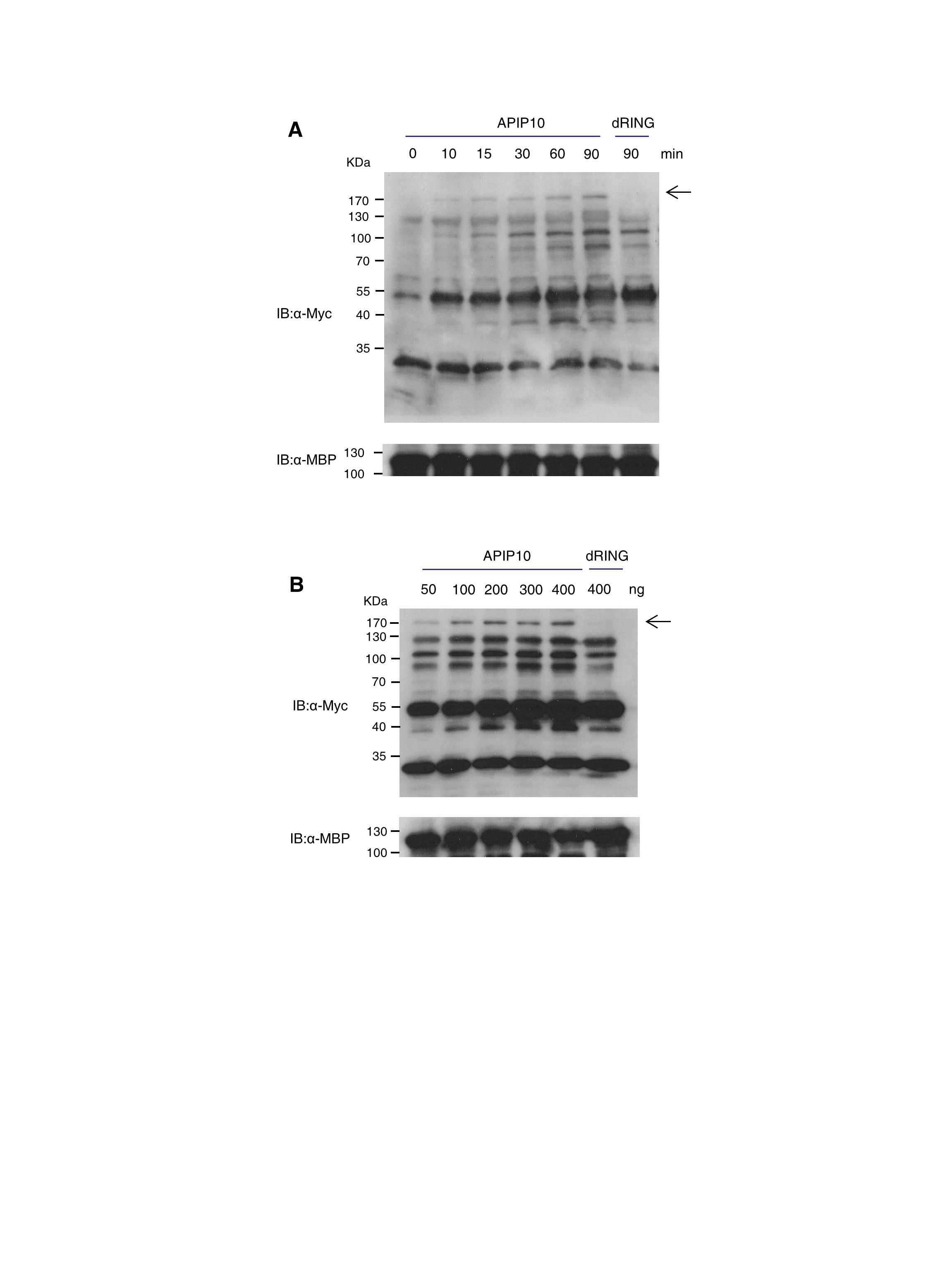

Supplement: S3 Fig — (A) Time-course assay of E3 ubiquitin ligase activity of APIP10. Time course ubiquitin ligase assay was performed with APIP10 for 90 min. dRING was included as a negative control. The protein amount of either APIP10 or APIP10 dRING (dRING) loaded in each lane was determined by western blot with anti-MBP antibody. (B) E3 ubiquitin ligase activity assay of APIP10 with different amount of E2 enzyme. E3 ubiquitin ligase assay was performed with APIP10 for 90 min with different amount of AtUBC10 (At5g53300) enzyme as indicated. A polyubiquitin band is indicated with an arrow. (TIF) [file ppat.1005529.s003.TIF]

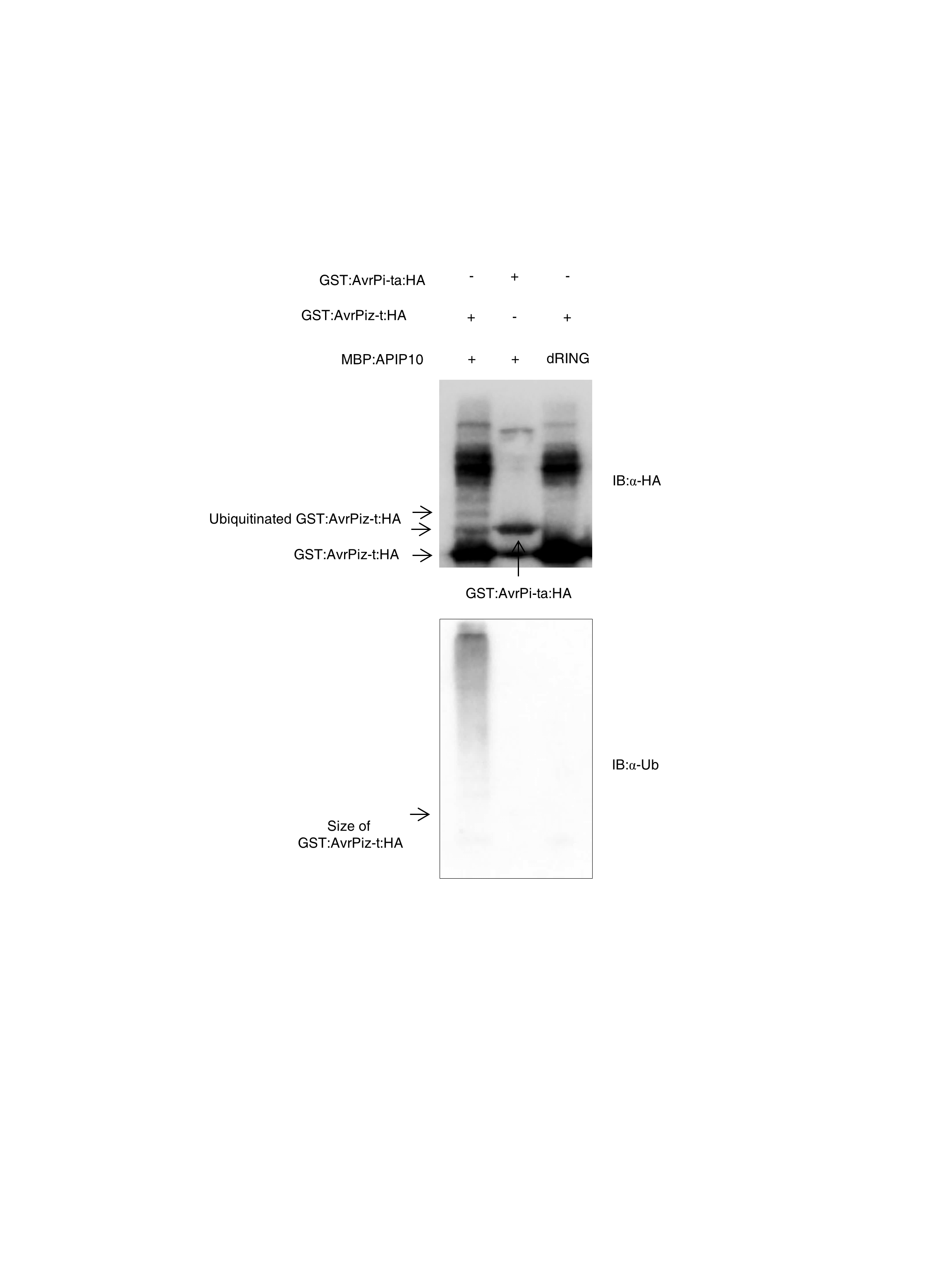

Supplement: S4 Fig — In vitro ubiquitination assay of GST:AvrPiz-t:HA by MBP:APIP10 was conducted with GST:AvrPiz-t:HA bound to glutathione agarose beads and washed five times with 1X PBST. Ubiquitinated GST:AvrPiz-t:HA was detected by western blot with the anti-HA antibody (upper panel) and the anti-Ub antibody after GST pulldown (lower panel). (TIF) [file ppat.1005529.s004.TIF]

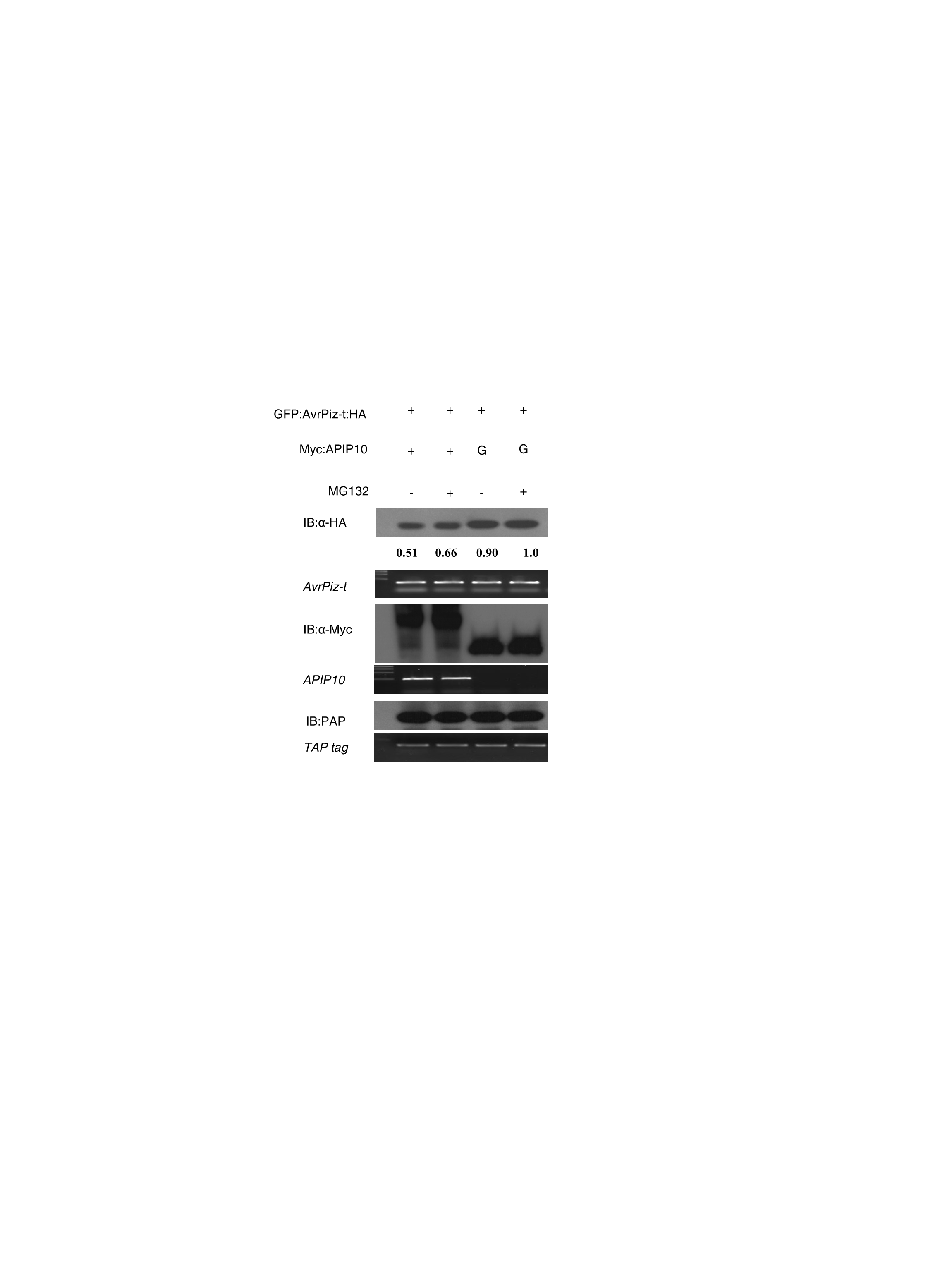

Supplement: S5 Fig — Myc:GFP was used as a negative control instead of Myc:APIP10 dRING. G, Myc:GFP. Experimental conditions were the same as those described in Fig 2A (TIF) [file ppat.1005529.s005.TIF]

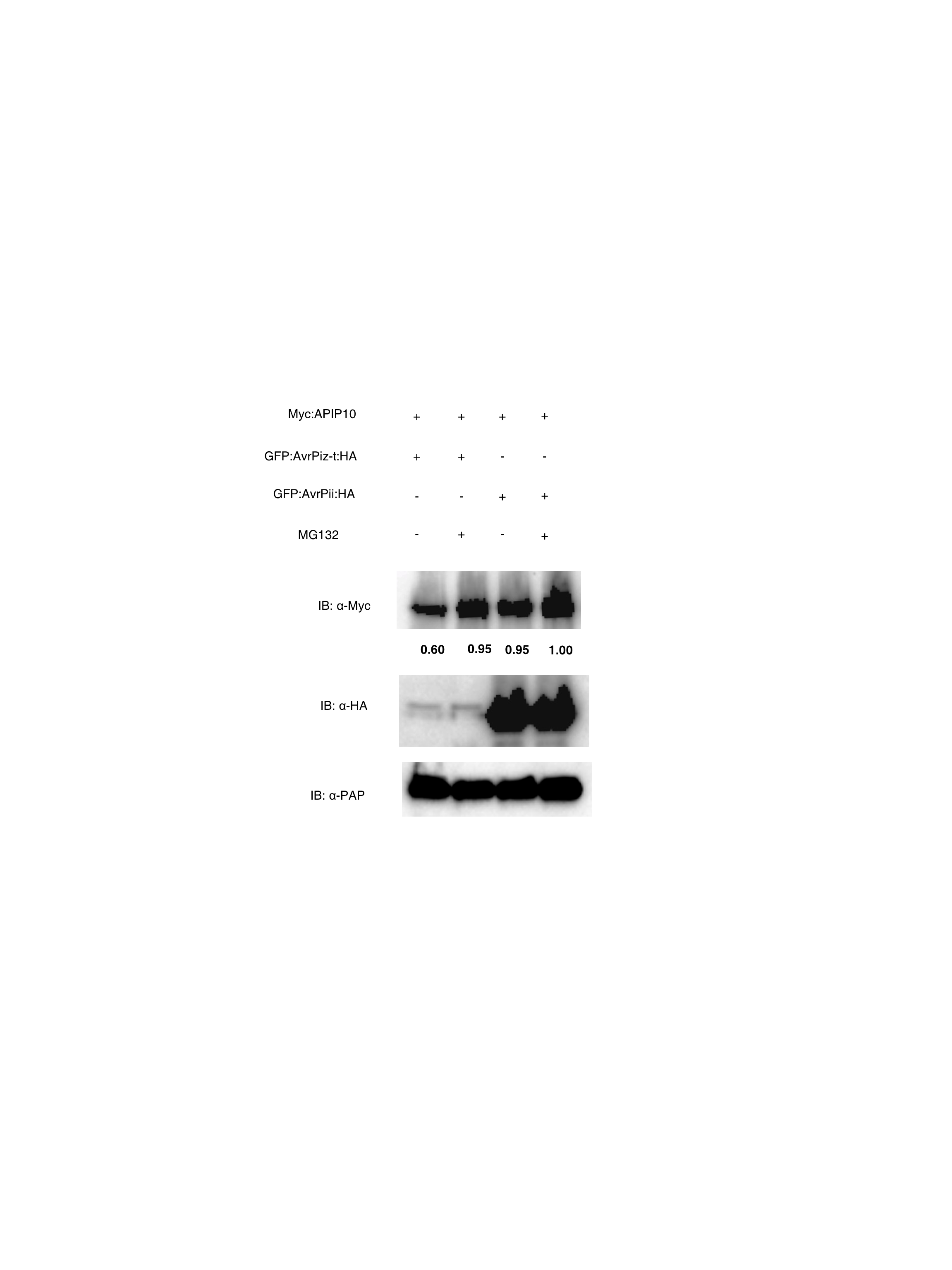

Supplement: S6 Fig — GFP:AvrPii:HA, an unrelated effector protein from M. oryzae, was used as a negative control. Tissues were harvested 3 days after infiltration. MG132 (50 μM) was infiltrated with DMSO as a control at 18 h before sampling. Experimental conditions were the same as those described in Fig 2B. (TIF) [file ppat.1005529.s006.TIF]

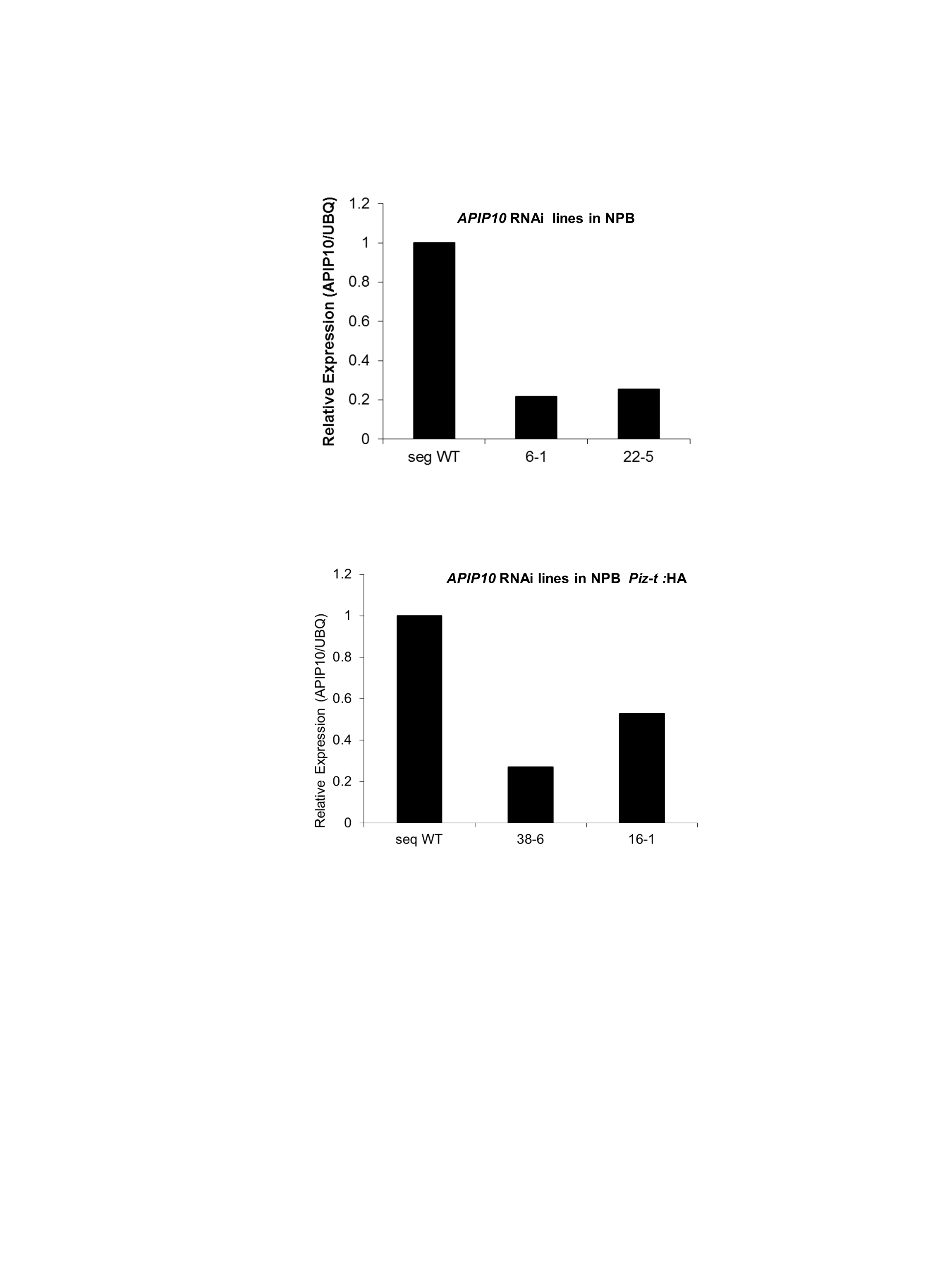

Supplement: S7 Fig — The transcript level of the ubiquitin (UBQ) was used for normalization. The analysis was repeated three times with similar results. (TIF) [file ppat.1005529.s007.TIF]

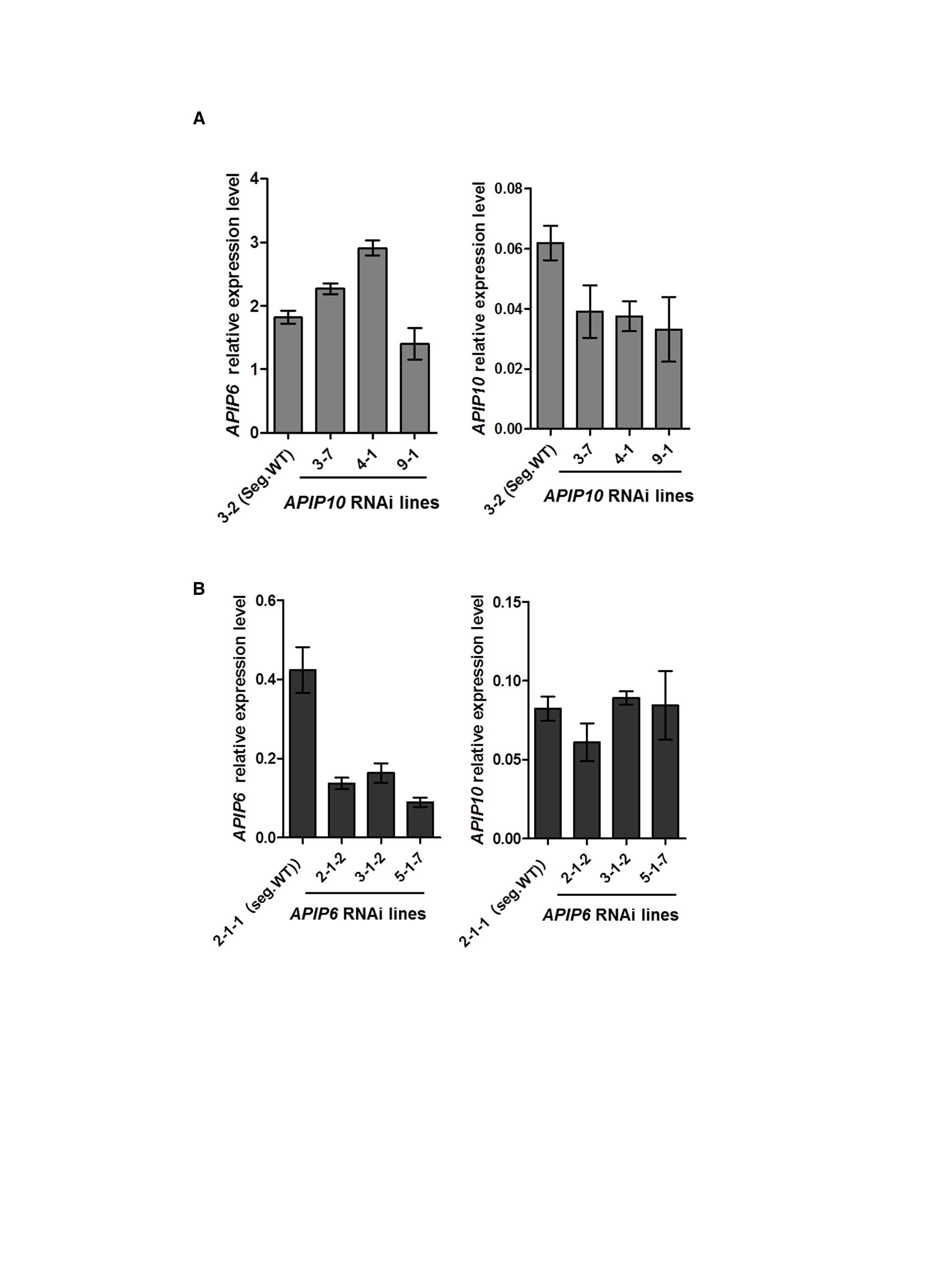

Supplement: S8 Fig — (A) Transcript levels of APIP6 and APIP10 in three different APIP10 RNAi lines. (B) Transcript levels of APIP6 and APIP10 in three different APIP6 RNAi lines (TIF) [file ppat.1005529.s008.TIF]

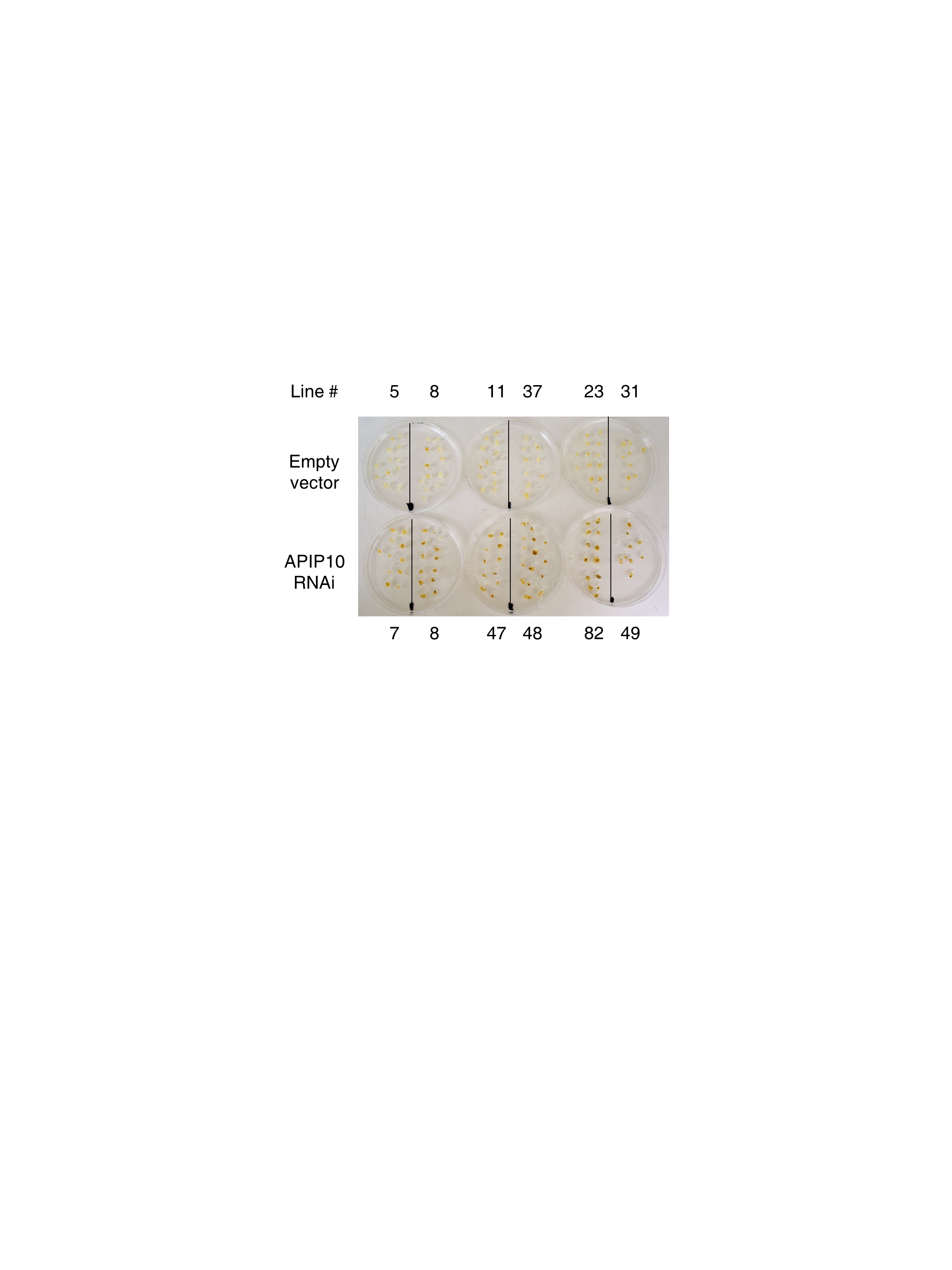

Supplement: S9 Fig — pCambia2300 empty vector was transformed into NPB Piz-t:HA background as a control of cell death phenotype observed in APIP10 silencing callus lines. (TIF) [file ppat.1005529.s009.TIF]

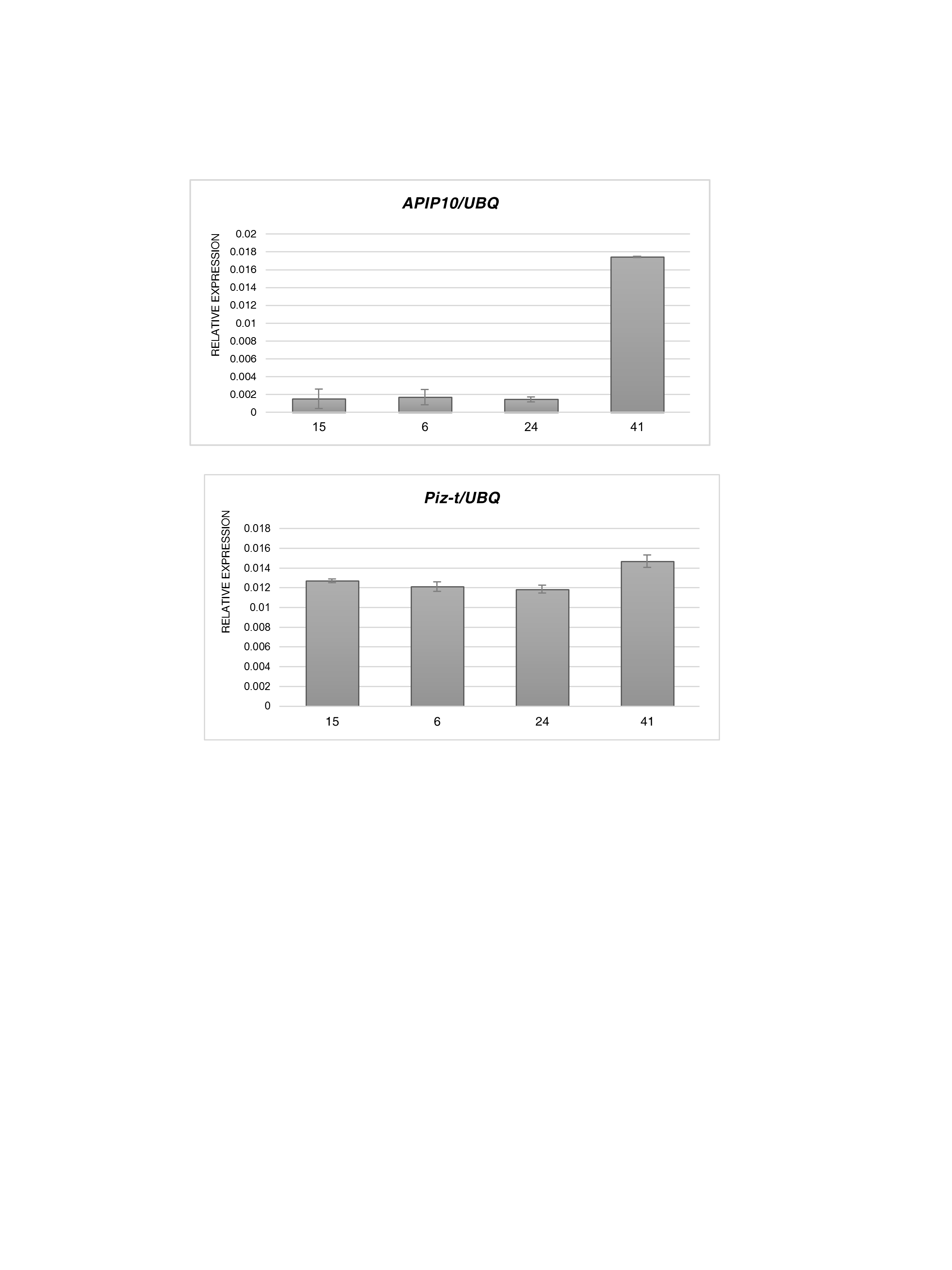

Supplement: S10 Fig — The expression level of APIP10 and Piz-t was relative to that of the rice ubiquitin (UBQ) gene. (TIF) [file ppat.1005529.s010.TIF]

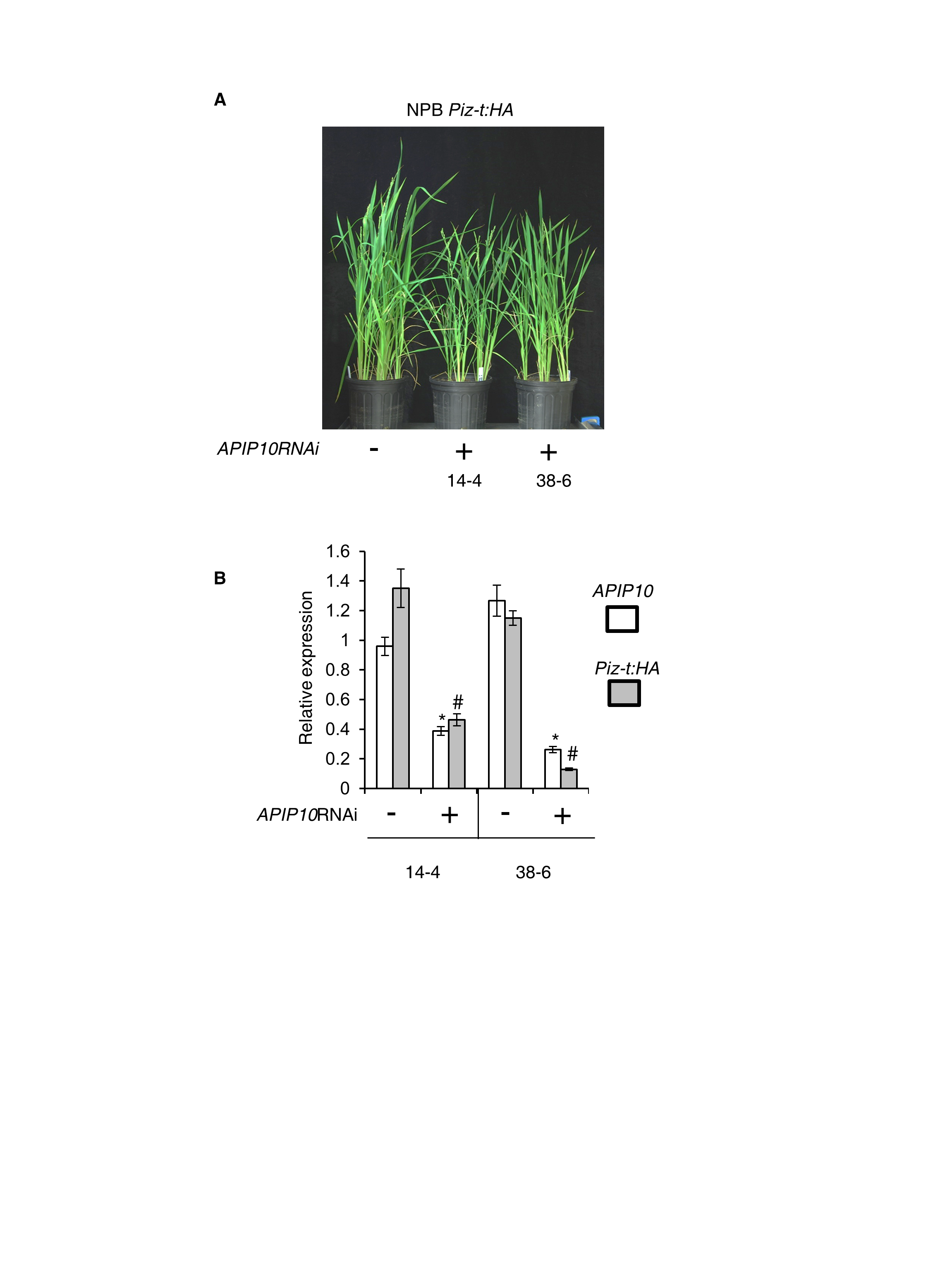

Supplement: S11 Fig — (A) Stunted growth of the NPB Piz-t:HA plants when APIP10 was silenced. (B) Transcript levels of APIP10 and Piz-t measured by qRT-PCR in 6-week old NPB Piz-t:HA APIP10RNAi plants. The transcript level of the ubiquitin (UBQ) was used for normalization. Data represent means and error bars indicate s.e.m. (* p<0.05 and # p<0.05; n = 3). (TIF) [file ppat.1005529.s011.TIF]

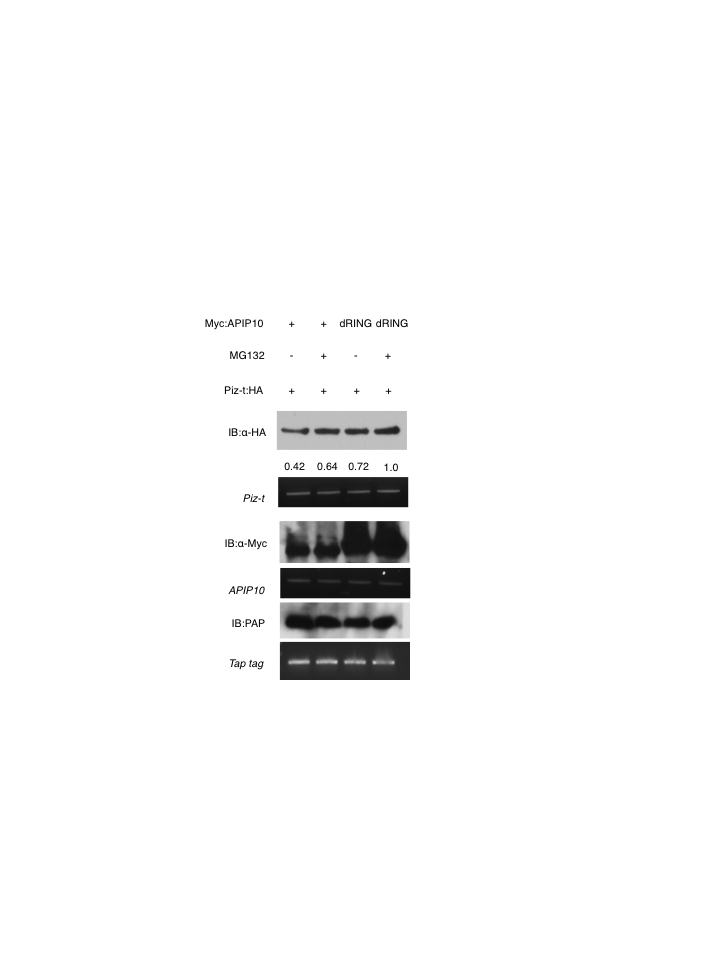

Supplement: S12 Fig — Piz-t:HA was co-expressed either with Myc:APIP10 or Myc:APIP10 dRING (dRING) in N. benthamiana. Agro-infiltrated tissues were harvested at 3 days after the infiltration and MG132 was treated 18 h before sampling. TAP tag was used as an internal control to normalize the agro-infiltration efficiency. Transcriptional level of each gene expression was determined by sqRT-PCR. (TIFF) [file ppat.1005529.s012.tiff]

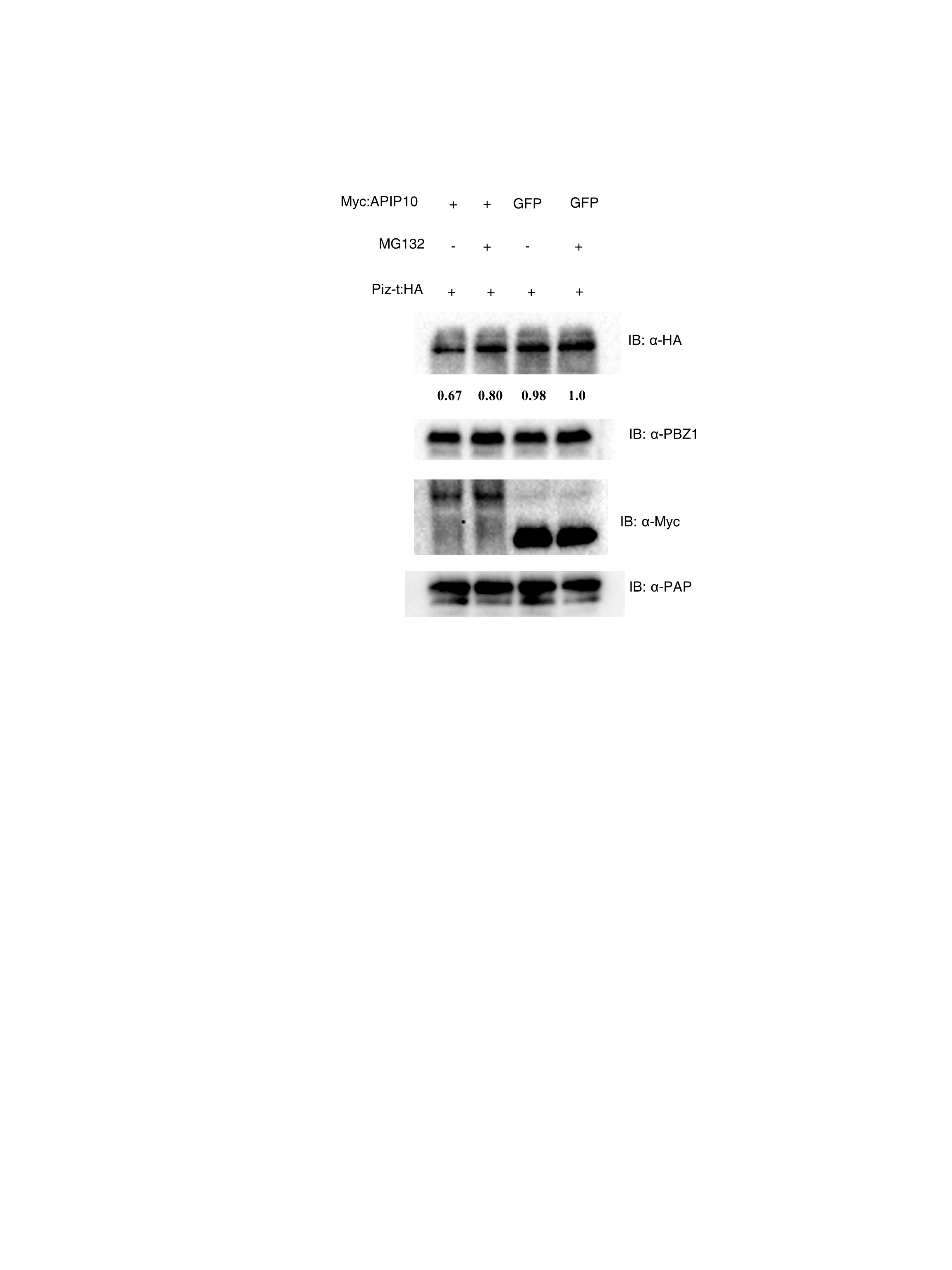

Supplement: S13 Fig — Degradation of Piz-t by APIP10 depends on APIP10 E3 ligase activity. Total protein from the inoculated rice plants was mixed with total protein from N. benthamiana in which either Myc:APIP10 or Myc:GFP was co-expressed with Taptag. PBZ1 or Taptag was used as a loading control for Piz-t and Myc:APIP10 or Myc:GFP, respectively. (TIF) [file ppat.1005529.s013.TIF]

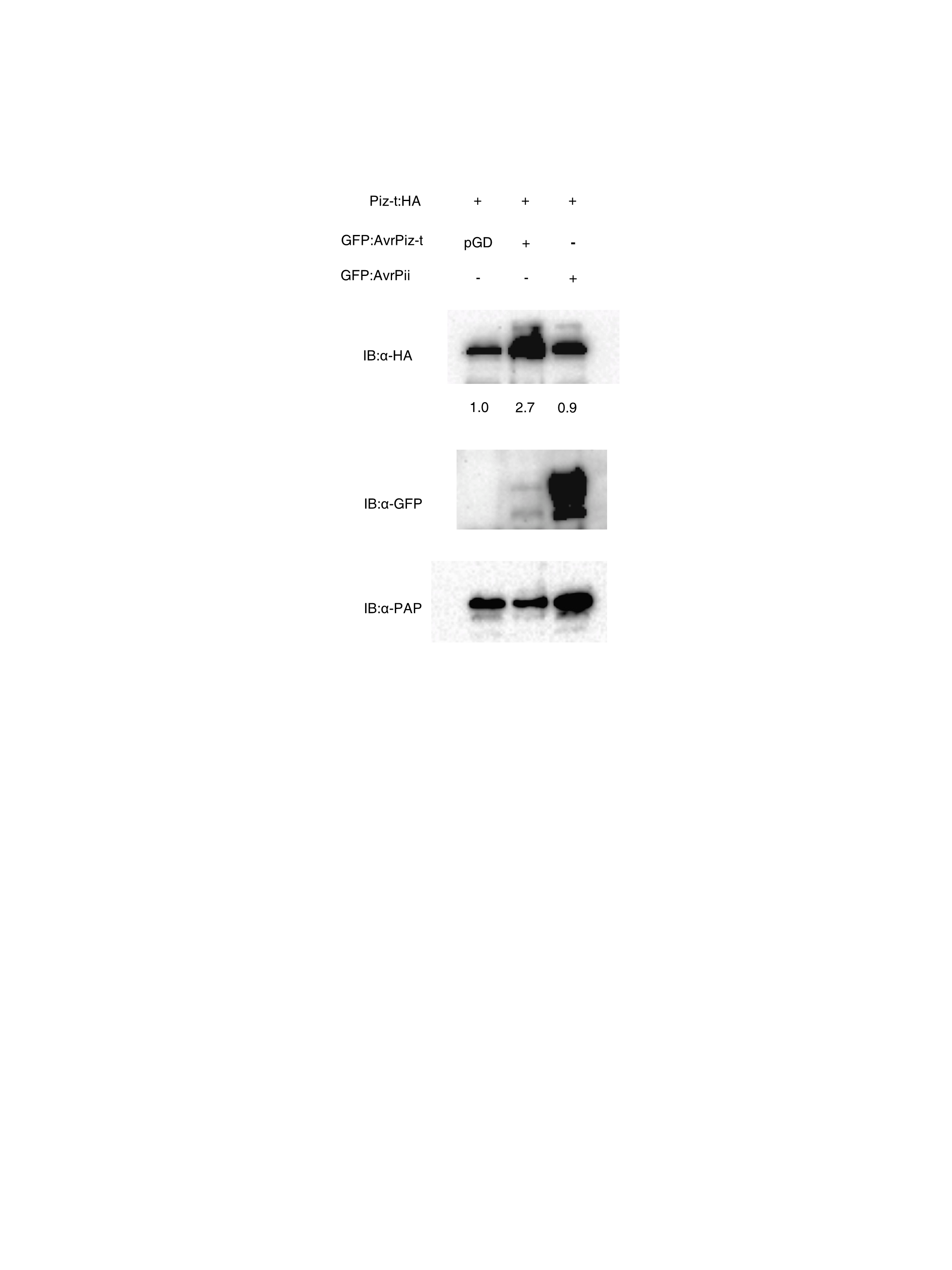

Supplement: S14 Fig — Co-expression of Piz-t:HA with GFP:AvrPiz-t:HA in N. benthamiana. The native promoter in the Piz-t genomic construct was replaced by the 35S promoter to express the Piz-t gene in N. benthamiana. Piz-t:HA was co-expressed either with pGD, GFP:AvrPiz-t, or GFP:AvrPii. Tissues were harvested at 4 days after agroinfiltration. TAP tag was expressed as an internal control to check the efficiency of agroinfiltration. (TIF) [file ppat.1005529.s014.TIF]

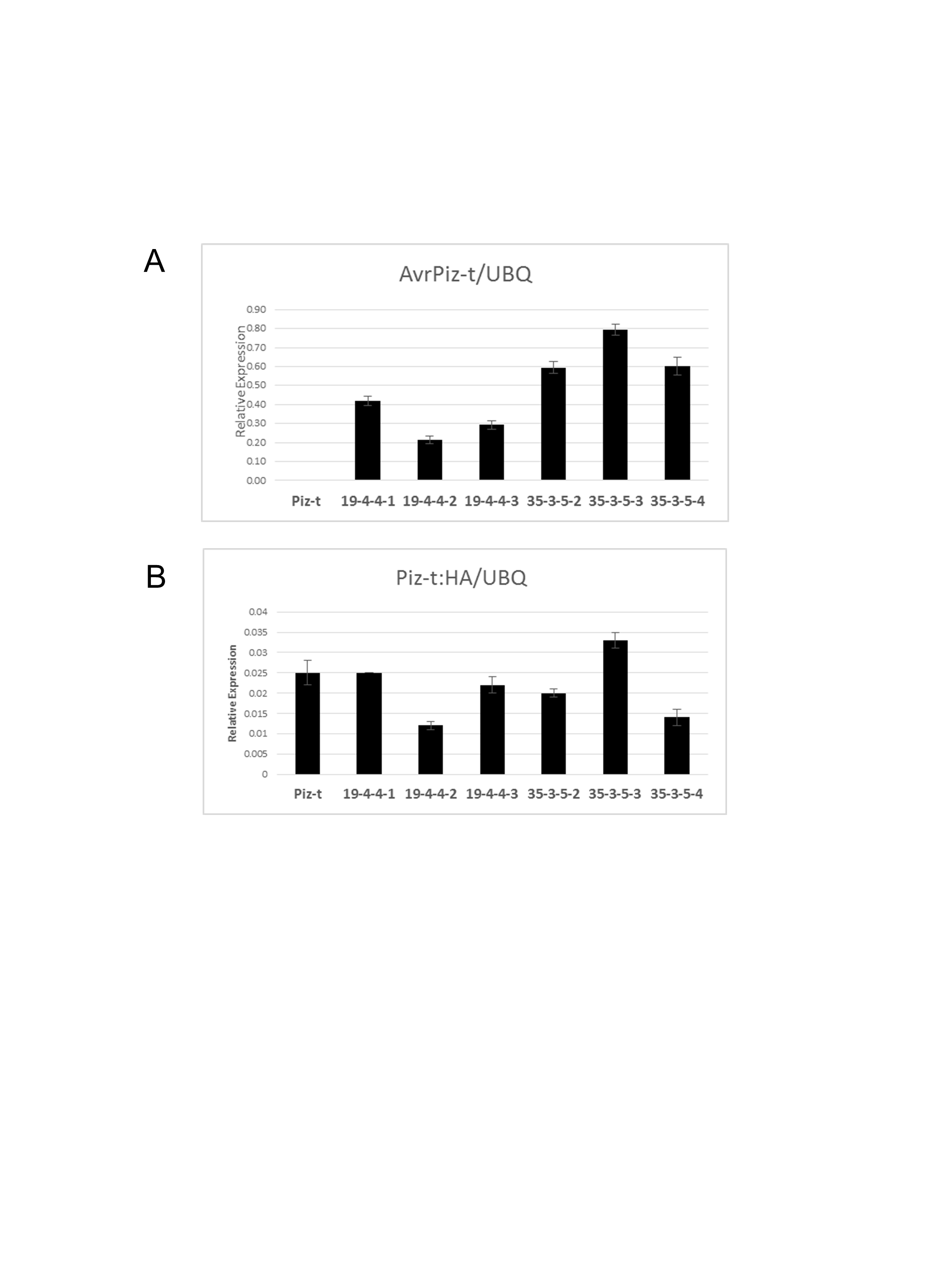

Supplement: S15 Fig — The samples were taken 24 h after the treatment. qRT-PCR with gene specific primer was performed, Ubiquitin (UBQ) transcript levels were used for normalization. Data are means of expression ratio and the error bars represent the s.e.m. (n = 2, P<0.05). (TIF) [file ppat.1005529.s015.TIF]
