# Supplementary material for: The E3 Ligase APIP10 Connects the Effector AvrPiz-t to the NLR Receptor Piz-t in Rice
Source: PLoS Pathog. 2016 Mar 31;12(3):e1005529. doi: 10.1371/journal.ppat.1005529 (PMC4816579; doi:10.1371/journal.ppat.1005529)
Supplement: S1 Table — (DOCX) [file ppat.1005529.s016.docx]

**Supplemental Table 1. Primers used in this study**

| Primers | Sequence | Purpose |
| --- | --- | --- |
| Apiz-t-HAF | 5’CCCGGATCCGAATTCAGCTTCGTACAATGC3’ | Cloning of AvrPiz-t into pXUN-HA vector |
| Apiz-t-HAR | 5’AGAGCTCGTCGACCCATTGGCGCTGAGCCTG3’ |  |
| APIP10-ProF | 5’GCCCGGATCCATGGCGACCTCCGGC3’ | Cloning of APIP10 in pMAL-c2x vector |
| APIP10-ProR | 5’CAAGGTCGACCTAATTTCTGATGCTGCTG3’ |  |
| AP10-5mycF | 5’CTTGAATTCCATGGCGACCTCCGGCGGCG3’ | Cloning of APIP10 in 5X Myc vector |
| AP10-5mycR | 5’AGAGGATCCTCAATACATTACAGCTTCCAT3’ |  |
| UBQ-SpeIF | 5’CCTCACTAGTATGCAGATATTCGTTAAGAC3’’ | Cloning of Ubiquitin in 5X Myc vector |
| UBQ-BamHIR | 5’GAAGGATCCCTAGCCTCCACGAAGGCGGA3’ |  |
| UBQ-BamHIF | 5’CTCGGGATCCATCGCTAGCAA’ | Cloning of 5X Myc ubiquitin in pGex-6p-1 |
| UBQ-SalIR | 5’CTAGAGTCGACCTAGCCTCCACGAAGGCGG3’ |  |
| dRING-F | 5’CTCAGCTGAACAACCTACACCAGTTTGCAGGTATTGTC3’ | Deletion of Ring Finger domain in APIP10 |
| dRING-R | 5’GACAATACCTGCAAACTGGTGTAGGTTGTTCAGCTGAG3’ |  |
| Pizt-CHA-F1 | 5’GGATCCCCTTCCCGAGCAGGAG3’ | Cloning Piz-t:HA in pCambia1305 |
| Pizt-CHA-R1 | 5’CCATGGTTAAGCGTAATCTGGAACATCGTATGGGTACCCGGGGCCAGCTTGAGCTGTG3’ |  |
| Pizt-CHA-F2 | 5’TACGCTTAACCATGGCGATCTCCTCCCCC3’ | Cloning Piz-t:HA in pCambia1305 |
| Pizt-CHA-R2 | 5’CTGCAGCCGATCTAGTAACATAG3’ |  |
| qAvrPiz-t_F | 5’AGACACTGGGGCACGATAAG3’ | qRT-PCR of AvrPiz-t and genotyping strains |
| qAvrPiz-t_R | 5’CCGGAGGAGAGAACATCAG3’ |  |
| *qOs*UBQ_F | 5’ AAGAAGCTGAAGCATCCAGC3’ | qRT-PCR of rice ubiquitin |
| q*Os*UBQ_R | 5’CCAGGACAAGATGATCTGCC3’ |  |
| APIP10RT_F | 5’AATTGAGAAGCTGGAGGAGG3’ | qRT-PCR of APIP10 |
| APIP10RT_R | 5’TGGTACCGAGAGAATTGTGC3’ |  |
| PAL-RT-F | 5’CTACCCGCTGATGAAGAAGC3’ | qRT-PCR of *PAL* |
| PAL-RT-R | 5’GAACCTTGTTCAGCTCCTCG3’ |  |
| qPiz-tHA_F | 5’TGTTGGAAAAGATAGAAATAGGCG3’ | qRT-PCR of *Piz-t:HA* |
| q3’HA_R | 5’cgtaatctggaacatcgtatgggt3’ |  |
| NAC4-RT-F | 5’TCCTGCCACCATTCTGAGATG3’ | qRT-PCR of NAC4 |
| NAC4-RT-R | 5’TTGCAGAATCATGCTTGCCAG3’ |  |
| KS4-RT-F | 5’TCGCATTGCGTGTGCAA3’ | qRT-PCR of *KS4* |
| KS4-RT-R | 5’TTGGAACTTCCGACATCGAAA3’ |  |
| *Os*UG_F | 5’TTCTGGTCCTTCCACTTTCAG 3’ | qRT-PCR of rice genomic ubiquitin |
| *Os*UG_R | 5’ACGATTGATTTAACCAGTCCATGA 3’ |  |
| *Mo*Pot2_F | 5’ACGACCCGTCTTTACTTATTTGG 3’ | qRT-PCR of *M. oryzae* retrotransposon *Pot2* |
| *Mo*Pot2_R | 5’AAGTAGCGTTGGTTTTGTTGGAT 3’ |  |
