# Supplementary material for: The E3 Ligase APIP10 Connects the Effector AvrPiz-t to the NLR Receptor Piz-t in Rice
Source: PLoS Pathog. 2016 Mar 31;12(3):e1005529. doi: 10.1371/journal.ppat.1005529 (PMC4816579; doi:10.1371/journal.ppat.1005529)
Supplement: S1 Methods — (DOCX) [file ppat.1005529.s017.docx]

**S1 Methods**

**Plant Material**

Rice (*Oryza sativa*) seeds were sterilized by immersion in 75% ethanol for 1 min followed by immersion in 2% sodium hypochlorite for 40 min. After the seeds were washed with sterile water, they were germinated on 1/2 MS medium for 1 week and then transferred to soil. Rice plants were maintained in a growth chamber at 26/22°C (light/dark, 12-h photoperiod) and 80% relative humidity.

**Yeast Two-hybrid Screening**

The ProQuest Two-Hybrid system (Invitrogen) was used for screening of the AvrPiz-t interacting proteins ([Vega-Sanchez et al., 2008](#_ENREF_6); [Park et al., 2012](#_ENREF_4)). Briefly, the full-length *NS-AvrPiz-t* was cloned in-frame into the bait vector pDBleu and was transformed into yeast strain Mav203. Yeast cells carrying pDBleu-*NS-AvrPiz-t* were transformed again with a rice cDNA library that was built with the prey vector pPC86 using mRNA isolated from the seedlings of rice line 75-1-127 (Vega-Sanchez, et al., 2008). Candidates selected on SD/-Leu-Trp+3AT medium were subjected to β-galactosidase assays for confirmation following the manufacturer’s protocol (Invitrogen).

**Plasmid construction**

In general, plasmids were constructed following the standard molecular manipulation procedures ([Sambrook and Russell, 2001](#_ENREF_5)). To express *AvrPiz-t* in rice protoplasts, we cloned the PCR-amplified *AvrPiz-t* fragment using the primer pair AvrPiz-t-HAF1/AvrPiz-t-HAR into the pXUN-CHA vector ([Chen et al., 2009](#_ENREF_1)).

*AvrPiz-t:CHA* was amplified with the primer pair AvrPiz-t-HAF1/AvrPiz-t-HAR2. After enzyme digestion with *BamH*I and *Xho*I, the DNA fragment was inserted into the *Bam*HI/*Sal*I site of pGex-6p-1 for protein expression in *E. coli* or into the *Bgl*II/*Sal*I site of pGD for infiltration in *N. benthanmiana* ([Goodin et al., 2002](#_ENREF_2)).

To express the APIP10 protein in *E. coli*, we cloned the *APIP10* fragment amplified with the primer pair AP10-ProF/AP10-ProR into the BamHI and SalI sites of pGex-6p-1 after the restriction enzyme digestion. To delete the RING finger domain of *APIP10*, we amplified two N- and C-terminal fragments of *APIP10* with the primer pairs AP10proF/dRING-R and dRING-F/AP10proR, respectively, and these two fragments were joined by DJ PCR.

For the expression of 5X *Myc-APIP10* in *N. benthamiana*, the *APIP10* fragment amplified with the primer pair AP10-5mycF/AP10-5mycR was cloned into the pCAMBIA2300-Ubi-Myc-OCS vector, resulting in *5X Myc:APIP10*. Then the 5X *Myc:APIP10* fragment was amplified with the primer pair 5mycF/APIP10-5mycR and inserted into the *Sac*I/*Bam*HI site of pGD after digestion with *Sac*I and *Bam*HI.

To generate 5X Myc:UBQ, we amplified the ubiquitin fragment with the primer pair of UBQ-*Spe*IF/UBQ-*Bam*HIR from rice cDNA. The fragment was first cloned into the pCAMBIA2300-Ubi-Myc-OCS vector, and then the 5X Myc:UBQ fragment was amplified with the primer pair UBQ-*Bam*HIF/UBQ-*Sal*IR. The fragment was cloned into pET28a for protein expression in *E. coli.*

For the construction of the *Tap* tag construct, the *Tap* tag fragment amplified from the pUbi.nc1300.ntapintron vector with the primer pair Taptag-F/Taptag-R was cloned into pGD for the expression in *N. benthamiana* as an internal control.

The *Piz-t:HA* construct for rice transformation was generated as follows. First, the *Pst*I-digested fragment containing a 3*'-*terminal part of the Piz-t genomic coding region and a *noparine synthase (Nos*) gene terminator from plasmid pCAMBIA1305-Piz-t ([Zhou et al., 2006](#_ENREF_8)) was inserted into the *Pst*I site of pBlueKSP. The resulting plasmid was designated pBPiz-t. Next, PCR fragments of the 3' end of Piz-t fused with the HA-tag sequence and of the *Nos* terminator were amplified using plasmid pCAMBIA1305-Piz-t as template with the primer pairs Pizt-CHA-F1/Pizt-CHA-R1 and Pizt-CHA-F2/Pizt-CHA-R2, respectively. An overlapping PCR was performed to fuse the two fragments with the primer pair Pizt-CHA-F1/Pizt-CHA-R2. The resulting PCR fragment was digested with *Bam*HI and *Pst*I, and then inserted into the *Bam*HI/*Pst*I digestion site to replace the original 3' end of the Pizt-Nos terminator fragment. The resulting plasmid was designated pBPizt-CHA. As a final step, the *Pst*I-digested fragment containing the 3*'-*terminal part of the Piz-t genomic coding region fused with the HA-tag sequence and the Nos terminator from plasmid pBPizt-CHA was inserted into the *Pst*I site; this fragment replaced the original 3*'-*terminal part of Piz-t and the *Nos* terminator fragment and generated plasmid pCAMBIA1305-Piz-t-CHA.

To express the *Piz-t:HA* in *N. benthamiana*, pCAMBIA1305-Piz-t-CHA was digested with either *Pst*I and *Nhe*I or *Hind*II and *Nhe*I. The resulting 3.2 kbp of HindIII/NheI and 4.5 kbp of NheI/PstI Piz-t:HA fragments were ligated into the HindIII/PstI site of pGD vector using three fragments ligation.

The primers used in this study for plasmid construction are described in Table S1.

**Protein Purification from *E. coli* and E3 Ubiquitin Ligase Activity Assays**

The full-length cDNAs of *APIP10* and *NS-AvrPiz-t* were expressed as MBP-tagged and GST-tagged proteins in *E. coli* strain Rosetta2 (DE3) and were affinity-purified with maltose (NEB) and glutathione matrix (Sigma), respectively. *In vitro* ubiquitination reactions were performed by adding 1 µg each of the MBP:APIP10 or MBP, 1 µg of GST:AvrPiz-t:HA, 40 ng of yeast E1 (Biomol), 100 ng of *Arabidopsis* E2 (AtUBC10), l μg of 5X Myc:UBQ, and 1.5 μl of 20X reaction buffer (1 M Tris HCl, pH 7.5, 40 mM ATP, 100 mM MgCl_2_, 40 mM DTT, 600 mM creatine phosephate, and 1 mg ml^-1^ creatine phosphokinase). The reaction was incubated at 30°C for 1.5 h in a 30-μl reaction volume before it was stopped by adding the SDS sample loading buffer and heating to 100°C for 5 min. Samples of the reactions were then separated in a 10 or 15% SDS–PAGE gel. Polyubiquitin bands were detected by immunoblot with the anti-Myc antibody (Sigma, USA) followed by chemiluminescence with an ECL kit (Promega, USA).

**Fungal Transformation**

The native promoter along with the signal peptide was amplified with the primer pair AvrPiz-t-FPro-MfeI/Apzi-t-SPlinkR from genomic DNA of rice blast field isolate KJ201. The product was then linked with *AvrPiz-t* and amplified with the primer pair-Apzi-t-SPlinkF/AvrPiz-tR by DJ-PCR ([Yu et al., 2004](#_ENREF_7)). The resulting P_AvrPiz-t_::AvrPiz-t was cloned into the pCX63 vector ([Kim et al., 2010](#_ENREF_3)) by the TA cloning method and the constructs were used for the generation of *M. oryzae* transformants expressing *AvrPiz-t* in protoplasts of field isolate RB22 via the PEG-mediated procedure.

***In vivo* Co-IP Assays**

For *in vivo* Co-IP assay, *Agrobacterium* strain GV3101 carrying expression vectors of *Myc:APIP10* and *GFP:AvrPiz-t:HA* was used to express proteins in *N. benthamiana* leaves via agroinfiltration. Two days after infiltration, *N. benthamiana* leaf tissues were harvested, and total proteins were extracted with a native buffer including 50 mM Tris-MES pH 8.0, 0.5 M sucrose, 1 mM MgCl_2_, 10 mM EDTA, 5 mM DTT, and plant protease inhibitor. A 10-µl volume of anti-HA agarose suspension (Sigma) was added to the protein samples, and the mixtures were kept at 4°C with head-to-tail shaking overnight. The samples were washed three times using 1X IP buffer according to the manufacturer’s instruction (Sigma). After 50 μl of 1X SDS loading buffer was added to each sample, the column was heated to 95°C for 5 min. About 15 μl of each sample was loaded to the protein gel for immunoblot analysis using anti-HA and anti-Myc antibodies.

**Supplemental References**

**Chen, S., Songkumarn, P., Liu, J., and Wang, G.L. (2009). A versatile zero background T-vector system for gene cloning and functional genomics. Plant Physiol 150, 1111-1121.**

**Goodin, M.M., Dietzgen, R.G., Schichnes, D., Ruzin, S., and Jackson, A.O. (2002). pGD vectors: versatile tools for the expression of green and red fluorescent protein fusions in agroinfiltrated plant leaves. Plant J 31, 375-383.**

**Kim, S., Hu, J., Oh, Y., Park, J., Choi, J., Lee, Y.H., Dean, R.A., and Mitchell, T.K. (2010). Combining ChIP-chip and expression profiling to model the MoCRZ1 mediated circuit for Ca/calcineurin signaling in the rice blast fungus. PLoS Pathog 6, e1000909.**

**Park, C.H., Chen, S., Shirsekar, G., Zhou, B., Khang, C.H., Songkumarn, P., Afzal, A.J., Ning, Y., Wang, R., Bellizzi, M., Valent, B., and Wang, G.L. (2012). The Magnaporthe oryzae effector AvrPiz-t targets the RING E3 ubiquitin ligase APIP6 to suppress pathogen-associated molecular pattern-triggered immunity in rice. Plant Cell 24, 4748-4762.**

**Sambrook, J., and Russell, D.W. (2001). Molecular cloning : a laboratory manual. (Cold Spring Harbor, N.Y.: Cold Spring Harbor Laboratory Press).**

**Vega-Sanchez, M.E., Zeng, L., Chen, S., Leung, H., and Wang, G.L. (2008). SPIN1, a K homology domain protein negatively regulated and ubiquitinated by the E3 ubiquitin ligase SPL11, is involved in flowering time control in rice. Plant Cell 20, 1456-1469.**

**Yu, J.H., Hamari, Z., Han, K.H., Seo, J.A., Reyes-Dominguez, Y., and Scazzocchio, C. (2004). Double-joint PCR: a PCR-based molecular tool for gene manipulations in filamentous fungi. Fungal Genet Biol 41, 973-981.**

**Zhou, B., Qu, S., Liu, G., Dolan, M., Sakai, H., Lu, G., Bellizzi, M., and Wang, G.L. (2006). The eight amino-acid differences within three leucine-rich repeats between Pi2 and Piz-t resistance proteins determine the resistance specificity to Magnaporthe grisea. Mol Plant Microbe Interact 19, 1216-1228.**
